# Water Oxidation by Molecular Catalyst Bilayers Self-Assembled on Bismuth Vanadium Oxide (BVO) Photoelectrodes

**DOI:** 10.1021/jacs.6c08907

**Published:** 2026-08-05

**Authors:** Paula Tris-Marzo, Pierpaolo Vecchi, Marcos Gil-Sepulcre, Gerald J. Meyer, Antoni Llobet

**Affiliations:** † Institute of Chemical Research of Catalonia (ICIQ), Av. Països Catalans 16, Tarragona 43007, Spain; ‡ Department of Chemistry, 2331 University of North Carolina at Chapel Hill, Chapel Hill, North Carolina 27599, United States; § Departament de Química Orgànica, Facultat de Química, 16781 Universitat de València, Av. Vicent Andrés Estellés 19, 46100 Burjassot, Spain

## Abstract

Sunlight-driven water splitting stands out as one of the viable strategies for the generation of a sustainable fuel. For this purpose, the coupling of proton reduction and water oxidation catalysis, with light absorbing materials is crucial. Here we develop a strategy that integrates a powerful molecular water-oxidation catalyst, based on oligomeric Ru-tda complexes (tda^2–^ is [2,2′:6′,2″-terpyridine]-6,6″-dicarboxylate), with a light absorbing BiVO_4_ material through self-assembly and formation of a bilayer. The self-assembly process also allows for the integration of a redox mediator, Ru-bpy (bpy is 2,2′-bipyridine), that was found to accelerate hole-transfer from the BiVO_4_ to the molecular catalyst leading to more efficient catalysis, as measured by transient absorption and intensity modulated photocurrent (IMPS) spectroscopies. With an applied potential of 0.82 V vs NHE (1.23 vs RHE) at pH 7 and 1 sun irradiation, photocatalytic water oxidation was observed with current densities of *ca*. 2 mA cm^–2^ and Faradaic efficiencies of 98%. This corresponds to turnover of over 23,500 per Ru unit (280,000 per Ru oligomer), ranking among the best reported at neutral pH. This new strategy yields photoelectrodes with enhanced stability and with very low catalyst loadings, that are expected to be versatile and easily implemented with other catalysts and semiconducting materials.

## Graphical abstract

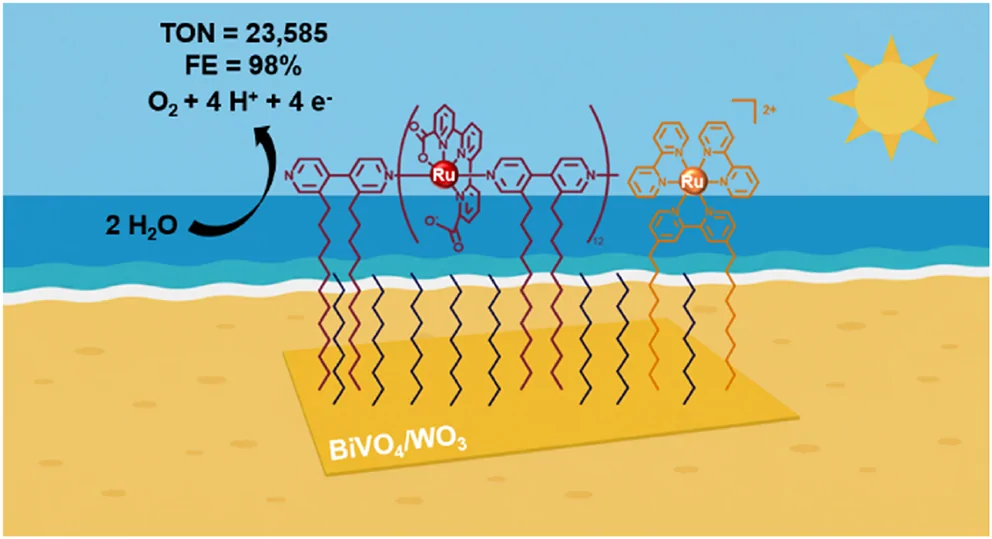

## Introduction

The massive use of fossil fuels has led to global warming with terrible environmental consequences. It is thus imperative to start the transition from fossil to sustainable and carbon neutral fuels.^1,2^ Solar-driven water splitting offers a direct route to transform sunlight and water into oxygen and hydrogen gas as a storable fuel.^3–6^ Decades of research on this topic indicate that water oxidation is the kinetic bottleneck that limits the efficiency and stability of water splitting photoelectrochemical cells. Further, efficient water oxidation will likely require both catalysts and light absorbing materials. Indeed, to date no single visible light absorbing material has been identified that efficiently catalyzes water oxidation. The integration of catalysts with light absorbing materials for water oxidation therefore represents a significant and unmet challenge. Importantly, if efficient and practical water photo-oxidation were realized, the electrons and protons generated could also be used to transform atmospheric CO_2_ into valuable liquid fuels similar to that found in green plants and algae.^7–9^

Water oxidation catalysis is particularly challenging due to (a) the high thermodynamic potential associated with the removal of 4 protons and 4 electrons from two water molecules; and (b) the mechanistically complex associated multielectron and multiproton processes that drive O–O bond formation and invariably slow down the associated kinetics.^10,11^ For solar applications, high performant water oxidation catalysts (WOCs) are essential for the achievement of practical water splitting photoelectrochemical cells (PECs), that also need to be effectively coupled to a light absorber enabling efficient transfer of photogenerated holes to the WOC.^12–15^ Thus, the interface between the light absorber and the molecular catalyst is a key element that determines the system’s performance and optimization requires a fundamental understanding of a number of factors that include semiconductor-catalyst electronic coupling, charge transfer kinetics, energy alignment, and photoelectrode stability. In this regard, a modular and synthetically versatile platform for constructing well-organized molecular-material hybrid photoelectrodes is highly desirable since many variations can be adopted whose study allows a judicious choice of the best self-assembled bilayer configuration.

Herein, we have tested the modular self-assembled bilayer approach to catalyst-semiconductor integration shown schematically in Figure 1A. This approach provides molecular-level control of the orientation of the catalysts with respect to the semiconducting light absorber, while maintaining low temperature processing conditions for catalyst integration.^16–20^ For this purpose, an alkylsilanolato group (SiO) with long alkyl chains was first bonded to a bismuth vanadate oxide (BiVO_4_) surface labeled as **BiVO**_**4**_ **|SiO**, that serves as a platform for the self-assembly of other molecular components that also contain long alkyl chains, through noncovalent H-interactions, Figure 1. The BiVO_4_ is the top layer in a “stack” of SnO_2_/WO_3_/BiVO_4_, whose conduction band energies provide a thermodynamic driving force for vectoral hole transfer to the water oxidation catalyst and electron transfer to the fluorine-doped tin oxide (FTO) substrate and the external circuit.

**Figure 1:**
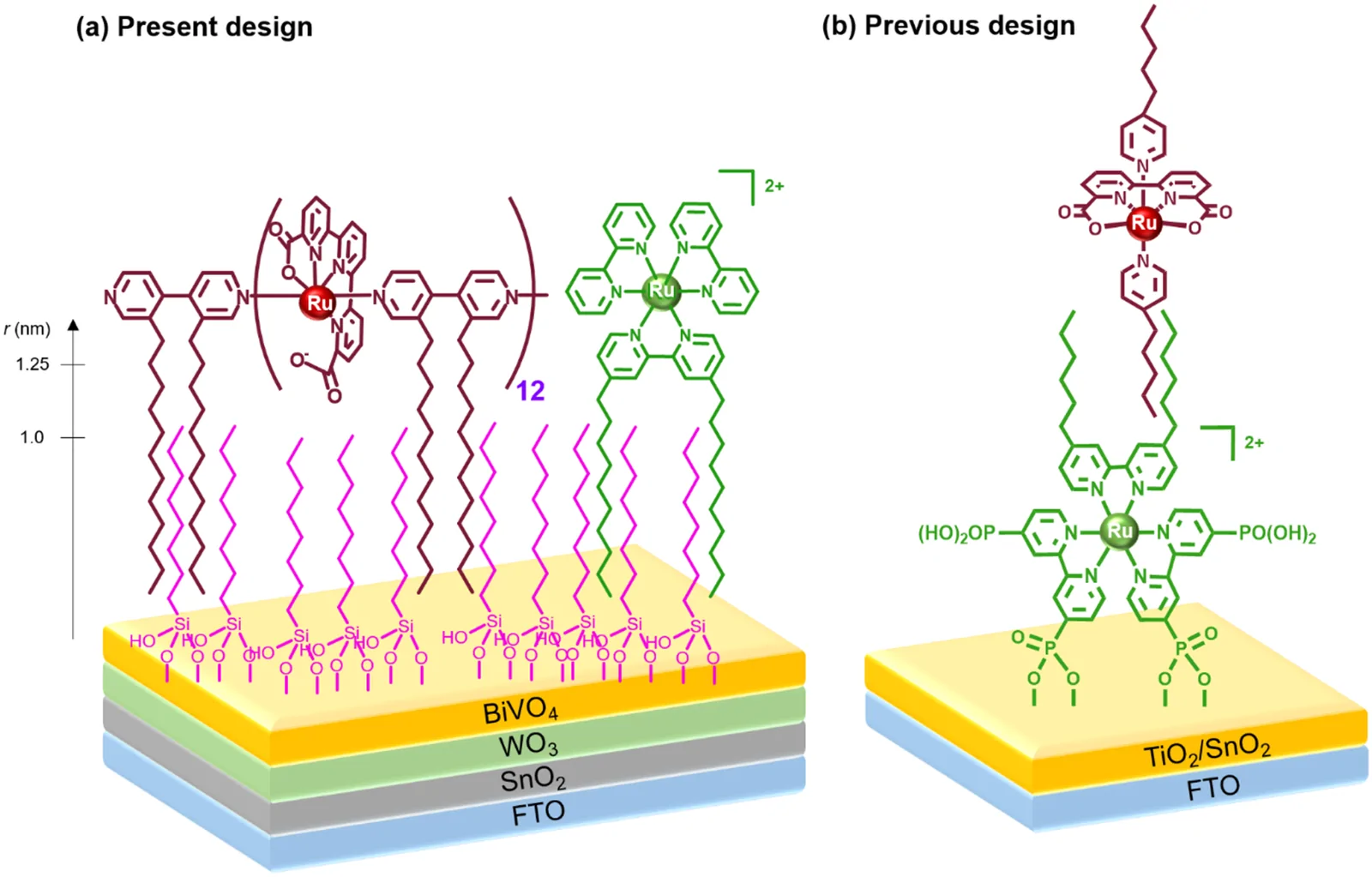
Hybrid photoelectrodes based on self-assembled bilayers. (A) drawing of a molecular water oxidation catalyst **Ru**_**Woc**_ (red) and an electron mediator **Ru**_**Med**_ (green) integrated with the alkylsilanolato (pink) modified BVO surface, that overall generates the **BVO|SiO|Ru**_**Woc**_-**Ru**_**Med**_ hybrid photoelectrode. (B) previous work based on the dye sensitized photoelectrosynthesis cell (DSPEC) approach using [Ru­(4,4′-(C_6_H_13_)_2_-bpy)­(4,4′-(PO_3_)_2_-bpy)_2_] as a dye (green) abbreviated as **Ru-b6bP** (**b6** is 4,4′-(C_6_H_13_)_2_-2,2′-bipyridine and **bP** is 4,4′-(PO_3_)_2_-2,2′-bipyridine), anchored to a TiO_2_ surface via alkyl–alkyl interactions to the water oxidation catalyst, [Ru­(bda)­(4-(C_5_H_11_)-py)_2_] (red), where bda is 2,2′-bipyridine-6,6′-dicarboxylate and 4-(C_5_H_11_)-py is 4-pentylpyridine abbreviated as **Ru-bda5**, to generate a related hybrid photoelectrode, **FTO|SnO**_**2**_ **|TiO**_**2**_ **|Ru-b6bP/Ru-bda5**.

As a water oxidation catalyst, an analogue of the oligomer **{[Ru** ^**II**^ **(tda)­(μ-4,4′-bpy)]**_**15**_ **(4,4′-bpy)}** where 4,4′-bpy is replaced by the bridging ligand 3,3′-dodecyl-4,4′-bipyridine (**b12**) generating **{[Ru** ^**II**^ **(tda)­(μ-b12)]**_**12**_ **(b12)}** abbreviated as **Ru**_**WOC**_, was selected. The latter contains 26 alkyl chains that readily self-assemble with the alkyl chains of the **BVO|SiO**, generating the new molecular hybrid photoanode **BVO|SiO|Ru**_**Woc**_ (see Figure 1A). As an electron mediator, an analogue of **[Ru­(bpy)**_**3**_ **]** ^2+^ where one of the bpy ligands is replaced by 4,4′-diundecyl-2,2′-bipyridine (**b11**), generating **[Ru** ^**II**^ **(bpy)**_**2**_ **(b11)]** ^2+^ abbreviated as **Ru**_**Med**_, was selected that contains 2 alkyl chains for self-assembly in the new molecular hybrid photoanode **BVO|SiO|Ru**_**Med**_. Using the same strategy, **Ru**_**Med**_ was self-assembled with **BVO|SiO|Ru**_**Woc**_ forming **BVO|SiO|Ru**_**Woc**_-**Ru**_**Med**_, that contains both the molecular water oxidation catalysts and the electron mediator. This photoelectrode is shown to provide sustained water oxidation catalysis with notably high photocurrent densities and stabilities.

## Results

### Preparation and Characterization of the Electrodes

The FTO/SnO_2_/WO_3_/BiVO_4_ material, referred to as **BVO** from now on, was used as a robust semiconductor support that provides a free energy gradient for electron migration from the BiVO_4_ to the external circuit and hole transfer to catalysts as has been described in the literature.^21–25^ The **BVO** surface was functionalized with long silanolato alkyl chains by hydrolysis of *n*-octyltrimethoxysilane as shown schematically in Figure 1 and labeled as **BVO|SiO** (see SI for more details). This material was then used as a substrate to self-assemble the electron relay and/or water oxidation catalyst via interdigitated alkyl–alkyl supramolecular interactions.^26^

The electrode morphology was characterized by Field Emission Scanning Electron Microscopy (FESEM), Atomic Force Microscopy (AFM) and by Energy dispersive X-ray spectroscopy (EDX) that provided a quantitative elemental analysis (Figures S5–S7). Further, the Ru content was measured by Inductively Coupled Plasma Mass Spectrometry (ICP-MS) that indicated a surface coverage of 0.50 nmols/cm^2^ for **BVO|SiO|Ru**_**Woc**_ and 13.6 nmols/cm^2^ for **BVO|SiO|Ru**_**Woc**_ **-Ru**_**Med**_.

Contact angles were also measured giving values of 44° and 81° for **BVO** and **BVO|SiO** respectively reflecting an expected increase in hydrophobicity due to the presence of the alkyl chains. The presence of the **Ru**_**Woc**_ catalyst in the **BVO|SiO|Ru**_**Woc**_ electrode increases the hydrophilicity about **BVO|SiO**, reducing the contact angle to 68° (see Figure S8).

The ultraviolet–visible (UV–vis) absorption spectrum of the Ru complexes **Ru**_**Woc**_ and **Ru**_**Med**_ in solution was measured in 2,2,2-Trifluoroethanol (TFE) and dichloromethane (DCM) respectively and reported in the SI (Figure S9). The spectra are dominated by π–π transitions in the ultraviolet region and by metal-to-ligand charge-transfer transitions in the range of 400–500 nm for **Ru**_**Med**_ ^27–29^ and 400–700 nm for **Ru**_**Woc**_.^30,31^ The UV–vis spectrum of **BVO|SiO|Ru**_**Woc**_ is reported in Figure 2a. The MLCT absorption of **Ru**_**Woc**_ was clearly observed at 510 and 555 nm confirming that once anchored the Ru complex maintains its identity. Note that a **BVO|SiO** thin film without the molecular catalyst was used as a reference.

**Figure 2:**
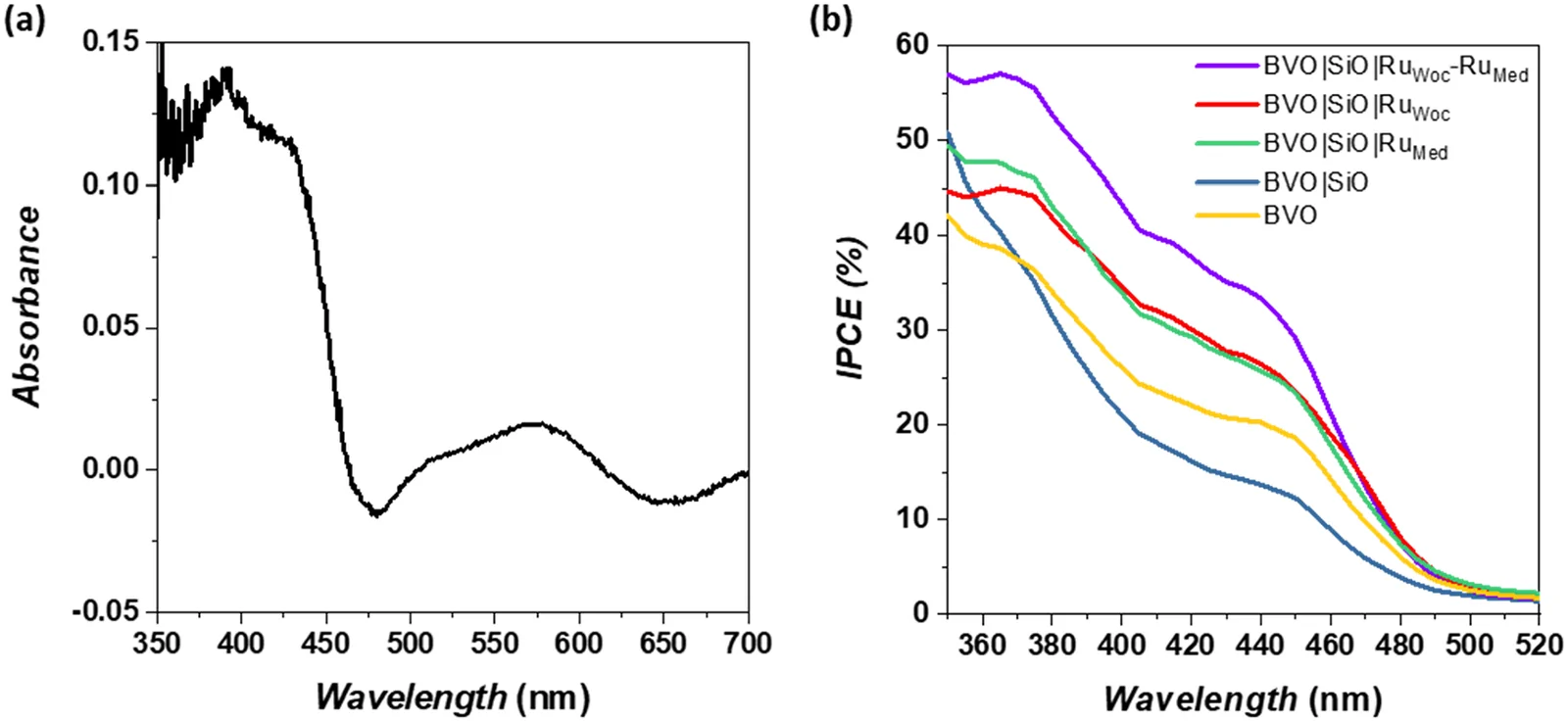
(a) The UV–visible absorption spectrum of **BVO|SiO|Ru**_**Woc**_, after subtraction of the **BVO|SiO** contributions. (b) IPCE measured at *E*_app_ = 0.82 V vs NHE (1.23 vs RHE) in a μ = 1.0 M pH 7 phbf aqueous solution on indicated samples.

The incident photon-to-current efficiency (IPCE, %) as a function of the excitation wavelength, i.e., a photocurrent action spectra, were measured from 520 to 350 nm under front-side illumination in a pH 7 phosphate buffer solution (μ = 1.0 M ionic strength) with a bias voltage *E*_app_ = 0.82 V vs. NHE (1.23 vs RHE). Light excitation at wavelengths longer than 520 nm did not result in the generation of a significant photocurrent for any of these photoelectrode materials. The results are displayed in Figure 2b. An unfunctionalized **BVO** photoelectrode displayed a measurable photocurrent with excitation wavelengths less than 500 nm with maxima in the photocurrent action spectra near 370 and 450 nm. Surface functionalization with the alkylsilanolato, **BVO|SiO**, resulted in *a* small decrease in the IPCE in the visible region. Catalyst **BVO|SiO|Ru**_**Woc**_ or mediator **BVO|SiO|Ru**_**Med**_ integration resulted in a significant increase in the photocurrent efficiency with an IPCE of 47% near 370 nm and 25% at 450 nm.^32^ When both the mediator and the catalyst were present **BVO|SiO|Ru**_**Woc**_ **-Ru**_**Med**_, the IPCE sharply increased reaching maxima of 56% at 370 nm and 35% at 450 nm. The coincidence of broad maxima near 375 and 450 nm in the photocurrent action spectra indicated that the photocurrent was generated by BVO light absorption. While we cannot rule out some excited state sensitization by **Ru**_**Med**_, we note that greater than 90% of the visible light is absorbed by the BVO rendering this as a minor pathway. The photocurrents were also quantified in the frequency domain using intensity modulated photocurrent spectroscopy (IMPS) and the results are shown in the Supporting Information.

### Transient Absorption Spectroscopy

Nanosecond transient absorption spectroscopic measurements were conducted after pulsed 355 nm excitation (2 mJ/cm^2^) of hybrid photoelectrodes at different applied potentials (*E*_app_). The goal was to understand the mechanism(s) by which photogenerated holes are captured by the catalyst and/or mediator. Key results are disseminated in Figure 3. Supra-bandgap excitation of the BVO material resulted in the appearance of a well-defined absorption band at 470 nm that has previously been assigned to photogenerated holes in BiVO_4_ and a weak absorption between 500 and 700 nm that has been assigned to intraband transitions.^33–38^ The fate of the photogenerated BiVO_4_ holes was of particular importance here and were quantified by monitoring the absorption kinetics at 470 nm. In all cases, the concentration of photogenerated holes was a maximum at the earliest 25 ns observation time and decayed cleanly to pre-excitation levels on a hundreds of microsecond time scale. Note that under these experimental conditions, the fate of holes in the other oxide materials as well as the redox chemistry of the molecular catalyst and mediators were not clearly observed due to the intense absorption of the BiVO_4_. Steady state absorption measurements made before and after these pulsed light excitation studies revealed no evidence for net photochemistry.

**Figure 3:**
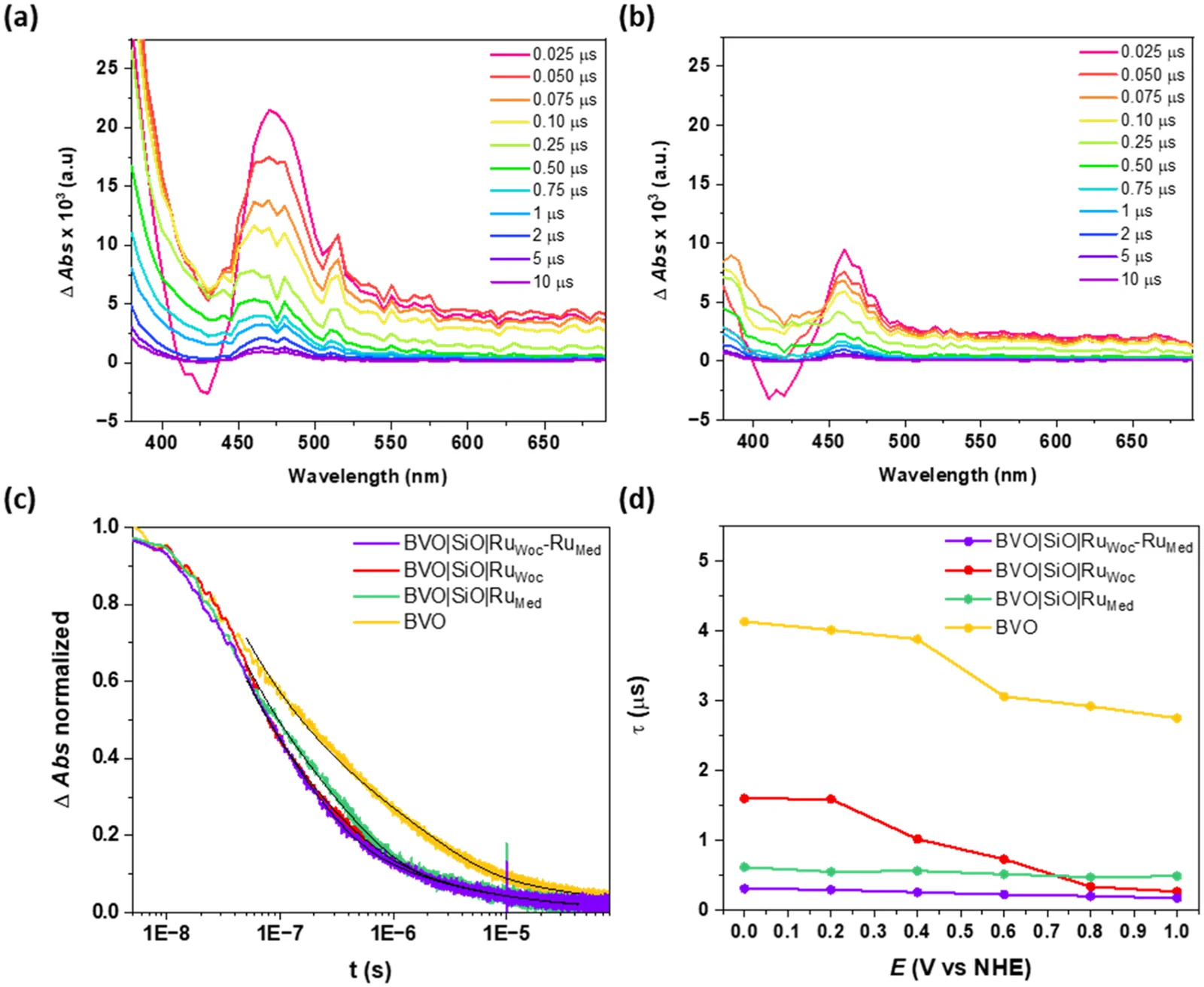
Transient absorption difference spectra measured at the indicated delay times after pulsed 355 nm (2 mJ/cm^2^) of: (a) **BVO** and (b) **BVO|SiO|Ru**_**Woc**_ both biased at 0.82 V vs NHE (1.23 vs RHE). (c) The normalized transient absorption change monitored at 470 nm after pulsed 355 nm light excitation with overlaid fits to eq 1 for **BVO** (yellow, τ = 2.92 μs), **BVO|SiO|Ru**_**Med**_ (green, τ = 0.47 μs), **BVO|SiO|Ru**_**Woc**_ (red, τ = 0.34 μs) and **BVO|SiO|Ru**_**Woc**_-**Ru**_**Med**_ (purple, τ = 0.20 μs). (d) Lifetime of the 470 nm absorption features, assigned to photogenerated holes, as a function of the applied potential for the same electrodes as in (c).

The absorption kinetics were well described by a previously reported power kinetic model (Figures S13 and S14), eq 1. The underlying mechanism was the subject of a previous WO_3_/BiVO_4_ photoelectrode study, wherein the power law decay component was assigned to second-order recombination through a distribution of characteristic rates.^39^ The exponential decay with a characteristic lifetime τ was assigned to first-order recombination of the trapped holes.

1 $$\Delta A\left(t\right)=A_{1}t^{-\alpha }+A_{2}\exp\left(-t/\tau \right)$$

For the unfunctionalized **BVO** with an applied potential *E*_app_ = 0.82 V, where water oxidation was most favorable, a lifetime of τ = 2.92 μs was measured, Figure 3a. Pulsed excitation of **BVO|SiO|Ru**_**Woc**_ resulted in a similar spectrum, but the amplitude of the 470 nm absorption was about half that measured with an unfunctionalized **BVO**, Figure 3b. This suggests that some hole transfer from BVO to the catalyst occurred on a sub-25 ns time scale. Furthermore, the 2.92 μs lifetime was about one-third of that measured on **BVO** providing clear evidence for hole transfer on longer time scales. Indeed, the inclusion of the catalyst and/or the redox mediator resulted in a significant decrease in the hole lifetime, Figure 3c. When the potential was stepped to more negative values where water oxidation was absent, the lifetimes increased to τ = 4.21 μs. In general, the hole lifetimes decreased as the applied potential was made more positive for **BVO** (yellow) and for **BVO|SiO|Ru**_**Woc**_ (red), Figure 3d.^40^ On the other hand, for the photoelectrodes containing the Ru mediator **BVO|SiO|Ru**_**Med**_ (green) and the **BVO|SiO|Ru**_**Woc**_ **-Ru**_**Med**_ (purple), the lifetimes are low, in the subμs time scale, and independent of the applied potential (see Figure 3d). This points to a fast transfer of holes to the redox mediator.

The valence band edge for BVO at pH 7 is approximately 2.5 eV,^41^ which provides a strong thermodynamic driving force for photogenerated holes to oxidize **Ru**_**Med**_^**II**^ in both its ground state (GS) and the excited state (ES),^42^ as well as the **Ru**_**Woc**_^**II**^ precursor.^31^ The thermodynamics of these reactions are shown below

2 $$\begin{array}{ll} {\mathrm{BVO}}^{*}+{{\mathrm{Ru}}_{\mathrm{Med}}}^{*}\to \mathrm{BVO}+{\mathrm{Ru}}_{\mathrm{Med}}^{\mathrm{III}} & \Delta E^{{}^{\circ}}=2.50+0.87=3.37\;V \end{array}$$

3 $$\begin{array}{ll} {\mathrm{BVO}}^{*}+{\mathrm{Ru}}_{\mathrm{Woc}}^{\mathrm{II}}\to \mathrm{BVO}+{\mathrm{Ru}}_{\mathrm{Woc}}^{\mathrm{III}} & \Delta E^{{}^{\circ}}=2.50-0.60=1.80\;V \end{array}$$

4 $$\begin{array}{ll} {\mathrm{BVO}}^{*}+{\mathrm{Ru}}_{\mathrm{Med}}^{\mathrm{II}}\to \mathrm{BVO}+{\mathrm{Ru}}_{\mathrm{Med}}^{\mathrm{III}} & \Delta E^{{}^{\circ}}=2.50-1.28=1.22\;V \end{array}$$

For an outer sphere electron transfer process as measured in the TAS, their reaction kinetics should be strongly dependent on their thermodynamic driving force. The TAS experiments presented in Figure 3, show that the ET between the BVO* and **Ru**_**Med**_^**II**^ is faster than for **Ru**_**Woc**_^**II**^, and hence the lifetime of the photogenerated holes lower. From a thermodynamic and interfacial kinetics perspective, the driving force for hole transfer is large and, in all cases, greater than the expected reorganization energy for interfacial electron transfer in the electric double layer.^43^ Hence these interfacial reactions are expected to fall in the Marcus kinetic inverted region. Since hole transfer to the mediator is the least thermodynamically favored it is expected to have the largest rate constant. Such rapid kinetics are consistent with the improved water oxidation efficiency that the mediator imparts. However, even in the absence of a large driving force, the Ru-bpy mediators are known kinetically to have remarkably large Ru­(III/II) self-exchange electron transfer rate constants^44^ and more rapid electron transfer to the BVO valence band alone could underlie the improved efficiency.

### The Photoelectrochemical Performance

The photoelectrochemical performance was evaluated under 1 sun (100 mW/cm^2^) illumination using a chopper (3 s on/off cycles) with linear sweep voltammetry (LSV) at a scan rate of 10 mV/s and by bulk electrolysis at *E*_app_ = 0.82 V vs NHE (1.23 vs RHE) for 1 h. The results are displayed in Figure 4a and 4b, respectively. The unfunctionalized **BVO** electrode (yellow trace) displayed photocurrent response of 1.2 mA/cm^2^ at *E*_app_ = 0.82 V vs NHE (1.23 vs RHE), consistent with values commonly reported for this material. Steady-state photolysis at this potential for 1 h revealed a relatively stable photocurrent of 0.8 mA/cm^2^ with a Faradaic Efficiency (FE) for O_2_ generation of only 64%, indicating that additional oxidations were occurring, most likely unwanted degradative processes. Compared to the alkyl functionalized electrode **BVO|SiO** the current density at *E*_app_ = 0.82 V decreased slightly and the FE after 1 h bulk electrolysis dropped to 40%, manifesting the hydrophobicity of the alkylsilanolato layer.

**Figure 4:**
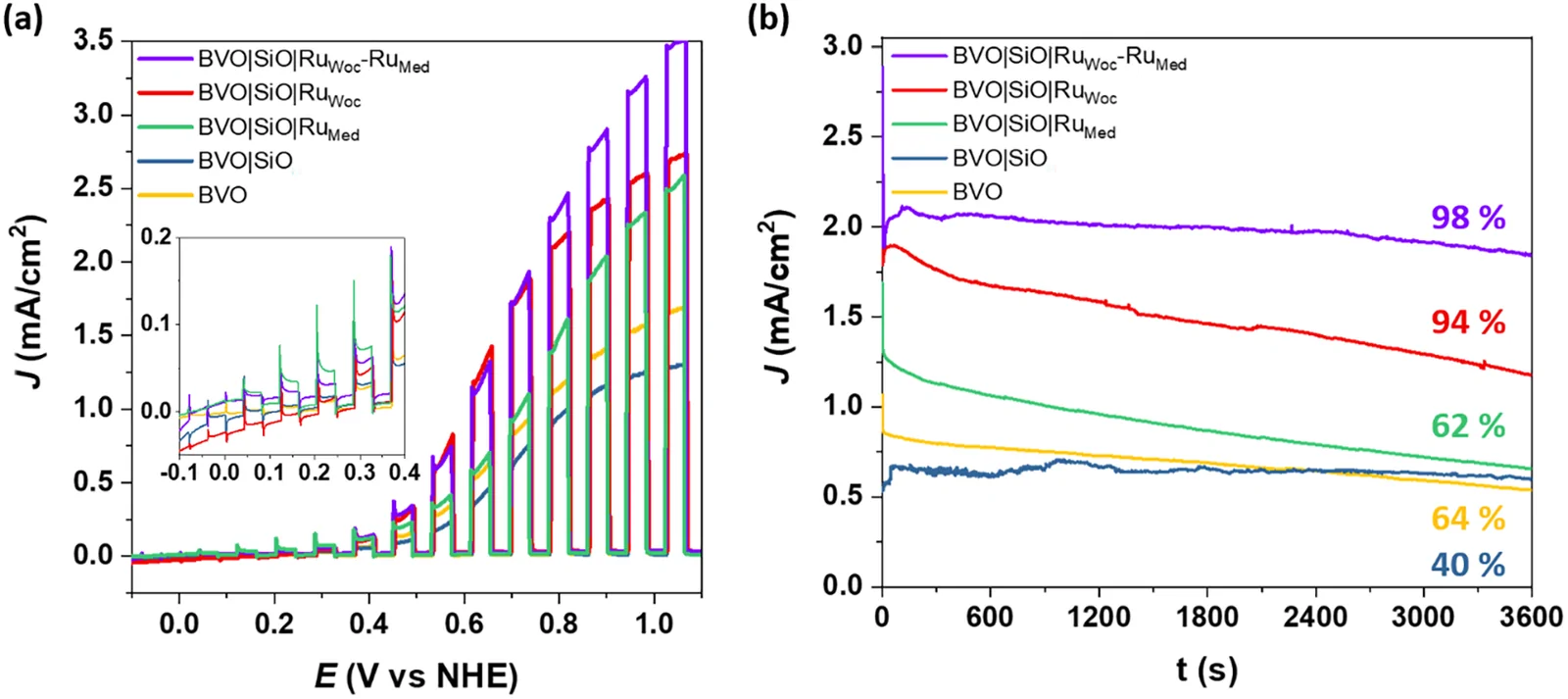
(a) Chopped linear sweep voltammetry at 10 mV/s irradiated with 1 sun (100 mW/cm^2^) for 3 s on/off cycles for: **BVO** (yellow trace), **BVO|SiO** (blue), **BVO|SiO|Ru**_**Med**_ (green), **BVO|SiO|Ru**_**Woc**_ (red) and **BVO|SiO|Ru**_**Woc**_-**Ru**_**Med**_ (purple) in a μ = 1.0 M pH 7 phbf aqueous solution. (b) Bulk photoelectrolysis photocurrent density measured at *E*_app_ = 0.82 V vs NHE (1.23 vs RHE) for 1 h for the same electrodes in (a).

In the presence of the Ru water oxidation catalyst, i.e., **BVO|SiO|Ru**_**Woc**_ (red trace), the current density at *E*_app_ = 0.82 V rose up to 2.1 mA/cm^2^ with a FE = 94%. The photocurrent generated as a function of *E*_app_ followed a similar pattern to that of the bare **BVO**, suggesting that in both cases the reactivity of the trapped states was dominant at *E*_app_ = 0.82 V, as indicated in Scheme 1 (top, left), and that at higher *E*_app_ when the BVO* trapped states were saturated then the current density leveled off.^39,45^ In the presence of the Ru mediator, **BVO|SiO|Ru**_**Med**_ (green trace), the photocurrent is enhanced up to 1.6 mA/cm^2^ at *E*_app_ = 0.82 V, but the FE was only of 62%, indicating that the mediator does not oxidize water to dioxygen. However, while the photocurrent of **BVO** started to level off at *E*_app_ = 0.82 V (yellow trace) that with the **Ru**_**Med**_ kept increasing with a nearly constant slope up to 1.1 V (green trace). This suggests that once the trapped holes were saturated at 0.82 V, the **Ru**_**Med**_ continued to enhance hole transfer from the valence band as depicted in Scheme 1 (top, right). Further, when both of the **Ru**_**Med**_ and the **Ru**_**Woc**_ were present as in **BVO|SiO|Ru**_**Woc**_-**Ru**_**Med**_ (purple trace), the current density at 0.82 V further increased up to 2.4 mA/cm^2^ with stable currents for about 1 h with a FE = 98% that represented a remarkable turnover number (TON) of 23,585 per Ru unit (or over 280,000 per Ru oligomer). The higher stability and higher FE of this electrode with regard to **BVO|SiO|Ru**_**Woc**_, points to the fact that the **Ru**_**Med**_ was also involved in the oxidation of the **Ru**_**Woc**_ across the bilayer that led to the formation of dioxygen, and thus manifesting the capacity of the **Ru**_**Med**_ to act as a photoelectron relay, enhancing the performance of the photoanode. The [Ru^III^(bpy)_3_]^3+^ (**Ru**_**Med**_^**III**^) mediator complex, generated from the reaction of BVO* and **Ru**_**Med**_ (eq 2) has a redox potential of 1.28 V, that is sufficiently high to drive the water oxidation catalytic process by **Ru**_**Woc**_ at pH 7, whose foot of the catalytic wave starts at 1.17 V.^31^

**Scheme 1:**
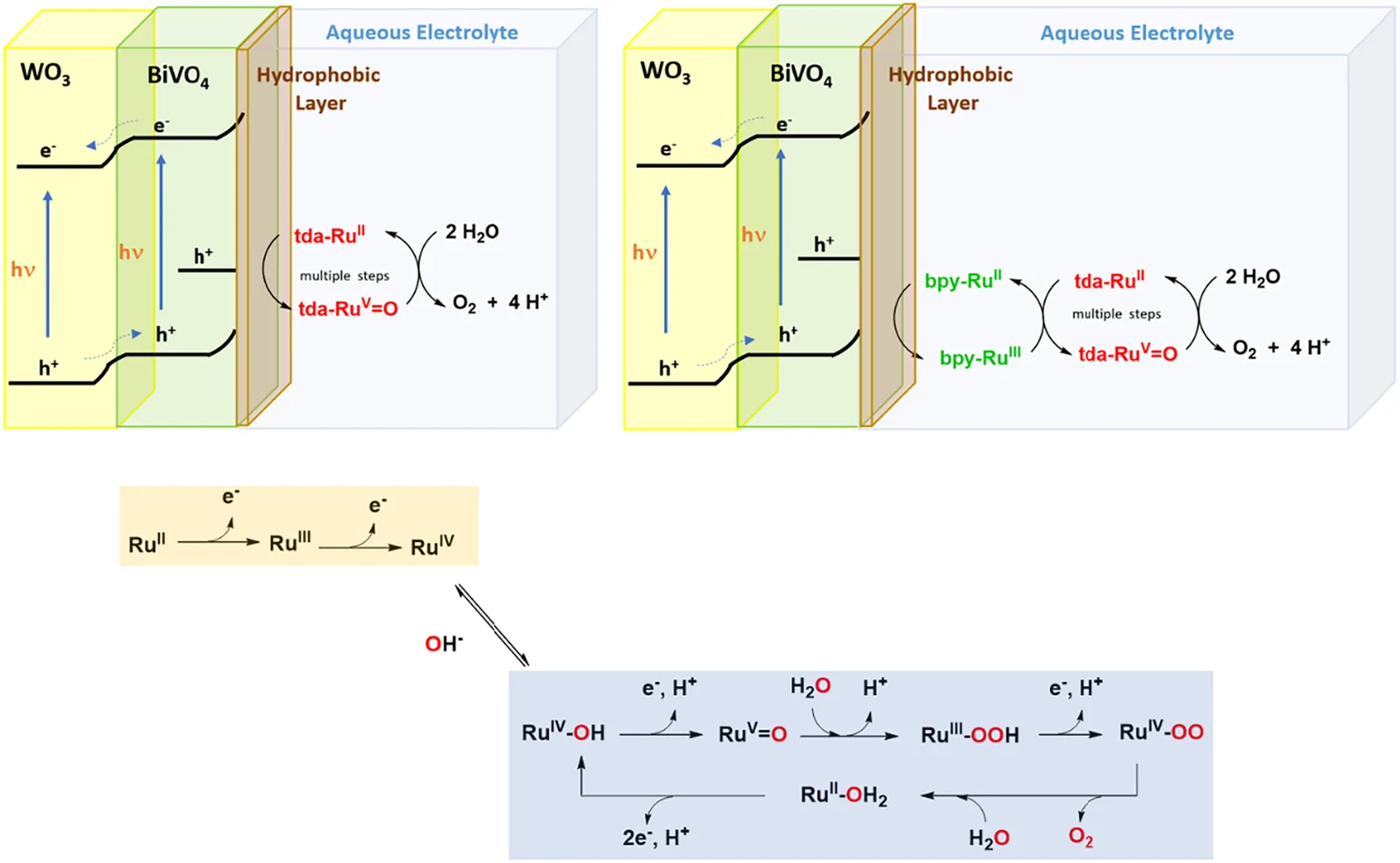
Top, Schematic View Showing the Reactions Involved in Light Induced Water Oxidation by **BVO|SiO|Ru**_**Woc**_ Electrode with the Trapped Holes (Left) and by **BVO|SiO|Ru**_**Woc**_-**Ru**_**Med**_ at High *E*_app_ Potentials with the Valence Band (Right). Bottom, the Reactions Associated with Water Oxidation Catalyst **Ru**_**Woc**_ Showing the Pre-equilibrium Reactions (Yellow Shaded) and the Species Associated with the Catalytic Cycle (Blue Shaded)^a^

a The **Ru**_**Woc**_ is depicted as a monomer with the tda and 4,4′-bpy ligands not shown for clarity.

Spectroscopic (UV–vis and XPS, Figures S10 and S19) and electrochemical techniques (Figure S20) before and after the bulk photoelectrocatalytic conditions described here, show that electrode stability is directly related to the photo hole transfer kinetics together with the FE. Thus, the combination of fast hole transfer kinetics using **Ru**_**Med**_, that minimizes BVO degradation process, together with the presence of the **Ru**_**Woc**_ that maximizes FE, are responsible for the robustness of the **BVO|SiO|Ru**_**Woc**_-**Ru**_**Med**_ photoelectrode.

At *E*_app_ > 0.82 V the photocurrent trend for **BVO|SiO|Ru**_**Woc**_-**Ru**_**Med**_ did not level off and instead follows a similar trend to that of **BVO|SiO|Ru**_**Med**_. This again suggested that **Ru**_**Med**_ plays a key mediator role at high potentials shuttling charge from the VB to the Ru­(III) species across the bilayer, that led to dioxygen formation as shown graphically in the bottom of Scheme 1. The generation of dioxygen by the Ru-tda complexes follows an activation process that is summarized in the yellow shaded part at the bottom of Scheme 1.^31^ Once the Ru^IV^ species is generated, it reacts with OH^–^ to form Ru^IV^(tda)­(OH) (axial ligands not written) that represents the gate to the water oxidation catalytic cycle as depicted in the blue shaded part at the bottom of Scheme 1.

The charge separation efficiency (CSE) was also quantified through intensity modulated photocurrent spectroscopy (IMPS) as has been previously described.^46^ Figure S17 shows the IMPS response for all the hybrid photoelectrodes measured over the same potential range under sinusoidally modulated 370 nm light excitation. Usually, the IMPS response of a photoelectrode plotted in a Nyquist plot is composed of a negative semicircle in the fourth quadrant reporting on charge separation and a positive semicircle describing the balance of charge transfer and recombination at the surface. For all the studied photoelectrodes, no recombination semicircle was observed, indicating low recombination and unitary transfer efficiency. The charge separation semicircle increased in size as the applied potential was made more positive, but also when the surface of BVO was modified with any of the Ru complexes, Figure S18 and Table S7.

Although speculative, the IMPS data suggests that alternative hole transfer process to the mediator and water oxidation catalysts occur besides those associated with the 475 nm absorption band assigned to trapped holes.^33–38^ Furthermore, larger CSE values for **BVO|SiO|Ru**_**Woc**_ **-Ru**_**Med**_ compared to **BVO|Si** indicate that more holes accumulate at the surface and are available to oxidize water, consistently with the rapid decrease of the trapped hole signal measured by TA when *a* WOC is at the surface.

## Discussion

A hybrid photoelectrode based on a bismuth vanadate, **BVO**, light absorber that was surface functionalized with alkylsilanolato groups, **BVO|SiO**, to yield a hydrophobic layer for the self-assembly of redox mediators and water oxidation catalysts that bear long alkyl chains. This self-assembly approach shares some similarities with prior work but differs significantly in the physical location and orientation of the catalyst relative to the light absorber. In addition, an inorganic oxide material was employed as the light absorber rather than a molecular chromophore, Figure 1B. The experimental data reported herein indicate that the **SiO** layer provides two benefits to the photoelectrode stability: (1) A hydrophobic interface that lowers the interfacial concentration of water and electrolyte that protects the bonds; and (2) A high density of alkyl chains that enable a net strong noncovalent integration through interdigitated alkyl–alkyl interactions. Notably, the **Ru**_**Woc**_ is a molecular oligomer bearing 26 alkyl chains, which can undergo multiple interactions with the alkyl chains on the **BVO|SiO** surface that may further benefit from a previously proposed rebound self-healing mechanism.^47^ As a result, the architectural design of the **BVO|SiO|Ru**_**Woc**_ **-Ru**_**Med**_ photoanode maintains photocurrent densities >2 mA/cm^2^ for an hour of illumination with nearly quantitative Faradaic efficiencies.

The alkylsilanolato coating in **BVO|SiO** situates the metal center of the mediator or catalyst roughly 1 nm from the BVO electrode surface where the valence band holes are photogenerated.^26^ Thus, electron transfer from the mediator, **Ru**_**Med**_, or the catalysts, **Ru**_**Woc**_, to the excited **BVO** occurs through an alkyl rich hydrophobic layer. Importantly, the characteristic absorption band previously assigned to trapped BVO holes enabled transient absorption characterization of this transfer with a mediator and/or a catalyst as a function of the applied potential. The intensity modulated photocurrent spectroscopy (IMPS) measurements also showed that the charge separation efficiencies were markedly higher at the more positive applied potentials as is discussed further below. Although not characterized herein, the electron transfer likely occurs via a quantum tunneling effect and/or a coherent through-bond tunneling along σ-bonds.^48–51^

Indeed, a key aspect of the design involves the presence of an electron transfer mediator to shuttle photogenerated holes from BVO* to **Ru**_**Woc**_. Previous studies for related systems have shown that the water oxidation rate can be enhanced by up to 5000-fold through the presence of an [Ru­(bpy)_3_]^2+^ type mediators,^52–54^ whose self-exchange (diffusion corrected) rate constant is 2.0 × 10^9^ M^–1^ s^–1^ and therefore ensures fast ET.^44,52,55^ For the **BVO|SiO|Ru**_**Woc**_ **-Ru**_**Med**_ electrode, the challenge is 2-fold and seeks to achieve: (1) fast ET from the **Ru**_**Med**_ to the BVO* through the hydrophobic alkyl rich bilayer that was clearly observed in the nanosecond absorption measurements; and (2) quantitative hole transfer from **Ru**_**Med**_ to the water oxidation catalyst **Ru**_**Woc**_, across the dynamic self-assembled bilayer. The latter was inferred from the increased photocurrent densities at high *E*_app_ as well as from the higher Faradaic efficiency measured in the case of the mixed electrode, **BVO|SiO|Ru**_**Woc**_ **-Ru**_**Med**_.

The key photoelectrochemical parameters reported here and for related BiVO_4_ photoanodes coated with molecular or oxide type heterogeneous water oxidation catalysts are gathered in Table 1 for comparative purposes. Entries 1–10 are based on a heterogeneous oxide-type catalyst,^56–63^ whereas entries 11–15 deal with molecular catalysts.^64^ Examination of Table 1 provides several interesting inferences. At an *E*_app_ = 0.82 vs NHE (1.23 vs RHE) under 1 sun irradiation in the presence of a buffer at pH 7, the highest photocurrent density reported was 3.9 mA/cm^2^ in entry 2,^65,66^ achieved using a relatively high catalyst loading of 320 nmols/cm^2^ on a conductive nickel support. In sharp contrast, the **BVO|SiO|Ru**_**Woc**_ electrodes (entry 14) achieved a comparable current density with about 600 times less catalyst, only 0.5 nmols/cm^2^. The molecular-level control of the spatial arrangement ensures that the catalysts are active and homogeneous across the BiVO_4_ surface bilayer, a behavior that is difficult to realize through the deposition of catalytic oxide materials. Other BiVO_4_ based photoanodes containing catalytic Mn, Fe or Co oxides studied at pH 7 (entries 1,^67^ 4,^68^ 9 and 10 ^39^) reported good photocurrent densities however the amount of catalyst is unknown. Significant photocurrent densities were also obtained in the absence of a buffer, as indicated in entry 5,^69^ using Na_2_SO_4_ as a supporting electrolyte and without a controlled pH during the photocatalytic experiment. Under more alkaline conditions of pH 8–9.3, entries 3,^70^ 6,^71^ 7 ^72^ and 8,^40^ higher current densities were generally obtained because the catalysis rate is known to increase with the OH^–^ concentration. However, most oxides are partly soluble under these conditions and thus will progressively leach into the electrolyte thereby decreasing the durability of the photoanode.^73–75^

**Table 1:** Key Photoelectrochemical Parameters Reported for BiVO_4_-Based Anodes Functionalized with Molecular Catalysts under 1-sun 100 mW/cm^2^ (1 sun) Illumination^a^

entry	photoanode	*J*_ph_ ^b^ (mA/cm^2^)	*E*_onset_ ^c^ (V vs RHE)	*E*_app_ ^d^ (V vs RHE)	*t* ^e^ (h)	FE (%)	loading^f^ (nmol/cm^2^)	electrolyte^g^	pH	ref
1	**BiVO**_**4**_ **|Mn**_**4**_ **O**_**4**_	2.50 (1.60)	0.60 (0.70)	1.23	2	-	-	0.2 M KPi	7	^67^
2	**BiVO**_**4**_ **/Al**_**2**_ **O**_**3**_ **|Ni**_**4**_ **O**_**4**_	3.90 (1.25)	0.36 (0.70)	1.23	0.02	96	320	0.2 M KPi	7	^65^
3	**BiVO**_**4**_ **|FeOOH**	1.70 (1.10)	0.22 (0.40)	0.50	1	95	-	0.1 M KPi	8	^70^
4	**BiVO**_**4**_ **|Co**_**4**_ **-POM**	2.10 (0.85)	0.20 (0.55)	-	-	90 (41)	1.15	0.5 M KPi	7	^68^
5	**BiVO**_**4**_ **|Co**_**4**_ **O**_**4**_	3.30	0.35	1.23	4	-	-	0.1 M Na_2_SO_4_ NO BUFFER	7	^69^
6	**BiVO**_**4**_ **|Co**_**4**_ **O**_**4**_	5.00 (1.60)	0.22 (0.40)	0.70	4	-	20	0.5 M KBi	9.3	^71^
7	**BiVO**_**4**_ **|CoPO**_**3**_	2.70 (1.25)	0.27 (0.40)	1.23	10	100	-	1.0 M KBi	9	^72^
8	**BiVO**_**4**_ **|Co**_**4**_ **O**_**4**_	3.30 (0.90)	0.18 (0.60)	1.00	22	100 (92)	-	0.5 M NaBi	8	^40^
9	**WO**_**3**_ **|BiVO**_**4**_ **|CoFeO**_**x**_	1.85 (0.90)	0.45 (0.80)	-	-	-	-	0.1 M NaPi	7	^39^
10	**WO**_**3**_ **|BiVO**_**4**_ **|CoFe-PB**	1.68 (0.90)	0.50 (0.80)	-	-	-	-	0.1 M NaPi	7	^39^
11	**FTO|SnO**_**2**_ **|TiO**_**2**_ **|Ru-b6bP/Ru-bda5** ^h^	2.2	--	1.02	0.5	73	--	0.1 M NaPi	7	^18^
12	**WO**_**3**_ **|BiVO**_**4**_ **|CNT|Ru-tda**	0.96 (0.88)	0.72 (0.81)	1.06	0.2	94 (97)	15	0.5 M NaPi	7	^77^
13	**WO**_**3**_ **|BiVO**_**4**_ **|Cu**_**Mac**_ ^i^	2.00 (1.60)	0.62 (0.72)	1.23	2.5	92 (70)	37	0.1 M NaPi	7	^64^
14	**BVO|SiO|Ru**_**Woc**_	2.20 (1.20)	0.63 (0.63)	1.23	1	93 (64)	0.50^j^	1.0 M NaPi	7	tw
15	**BVO|SiO|Ru**_**Woc**_ **-Ru**_**Med**_	2.44 (1.20)	0.53 (0.63)	1.23	1	98 (64)	13.6^k^	1.0 M NaPi	7	tw

a The values in parentheses are for **BiVO**_**4**_, **WO**_**3**_ **|BiVO**_**4**_ or BVO in the absence of catalyst.

b J_ph_ (mA/cm^2^) is the photocurrent at 1.23 V vs RHE; In parentheses is the performance of the electrode in the absence of catalyst.

c V_onset_ (V vs RHE) is the onset potential;

d V (V vs RHE) is the potential exerted on stability test;

e Time (h) is the time of stability test;

f Amount of catalyst (nmol/cm^2^) anchored onto the photoanode;

g KPi: potassium phosphate, KBi: potassium borate, NaBi: sodium borate;

h **FTO/SnO**_**2**_ **/TiO**_**2**_ **|Ru-bda5,Ru-b6bP**, where **Ru-bda5** is [Ru­(bda)­(4-(C_5_H_11_)-py)_2_] (bda is 2,2′-bipyridine-6,6′-dicarboxylate and 4-(C_5_H_11_)-py is 4-pentylpyridine) and **Ru-b6bP** is [Ru­(4,4′-(C_6_H_13_)_2_-bpy)­(4,4′-(PO_3_)_2_-bpy)_2_] (**b6** is 4,4′-(C_6_H_13_)_2_-2,2′-bipyridine and **bP** is 2,2′-bipyridine-4,4′-diphosphonate).

i Mac is 15,15-bis­(6-(thiophen-2-yl)­hexyl)-8,13-dihydro-5H-dibenzo­[*b*,*h*]­[1,4,7,10]­tetraazacyclotridecine-6,7,14,16­(15H,17H)­tetraone.

j 0.5 nmols of Ru/cm^2^ that corresponds to 0.04 nmols of oligomer/cm^2^.

k overall 13.6 nmols of Ru/cm^2^ that corresponds to a ratio of 0.5/13 of Ru-tda repeating unit/**Ru**_**Med**_.

An example based on the self-assembled bilayer methodology has been reported by Concepcion et al.^18^ (See Figure 1B; Table 1, entry 11)^76^ where the light absorber was [Ru­(4,4′-(C_6_H_13_)_2_-bpy)­(4,4′-(PO_3_)_2_-bpy)_2_], **Ru-b6bP**, and using a Ru-bda type of water oxidation catalyst [Ru­(bda)­(4-(C_5_H_11_)-py)_2_], **Ru-bda5**, which together generates an **FTO|SnO**_**2**_ **|TiO**_**2**_ **|Ru-b6bP/Ru-bda5** photoanode. Good photocurrent densities were measured with this hybrid photoelectrode that were comparable to **BVO|SiO|Ru**_**Woc**_ **-Ru**_**Med**_ (entries 11, 14, 15). However, the stability at neutral and higher pH values was greatly compromised by the bulky **Ru-b6bP** complex and by the relatively low number of alkyl–alkyl interactions of the bpy ligands to accommodate the **Ru-bda5** water oxidation catalyst.

Additional hybrid photoelectrodes abbreviated **WO**_**3**_ **|BiVO**_**4**_ **|CNT|Ru-tda** and **WO**_**3**_ **|BiVO**_**4**_ **|Cu-Mac** have been reported with key performances presented in entries 12 and 13, respectively. For **BVO|CNT|Ru-tda**, a mononuclear Ru-tda water oxidation catalyst was supported on carbon nanotubes (CNT) via π–π supramolecular interactions forming **CNT|Ru-tda**, which was then drop casted on *a* light absorbing **WO**_**3**_ **|BiVO**_**4**_ electrode. Good photocurrent densities in the range of 1.0 mA/cm^2^ at *E*_app_ = 0.82 V vs NHE (1.23 vs RHE) and good stability were reported. On the other hand, entry 13 shows the performance of a BVO photoelectrode containing **Cu-Mac**, a molecular water oxidation photoelectropolymerized at the BVO surface. The resulting **BVO|Cu-Mac** hybrid material displays good current densities in the range of 2.0 mA/cm^2^ at *E*_app_ = 0.82 V vs NHE (1.23 vs RHE) with good stability but needs more than 70 times more mass than **BVO|SiO|Ru**_**Woc**_ to reach comparable current densities (entry 14).^64^

## Conclusions

Hybrid photoanodes based on supramolecular self-assembled bilayers (SABs), in which a **BVO** light absorbing semiconductor was functionalized with alkylsilanolato groups **BVO|SiO**, that act as platform to self-assemble water-oxidation catalysts and mediators. The synthetic versatility of this self-assembly approach allowed mixed hybrid molecular materials where both the **Ru**_**Woc**_ and **Ru**_**Med**_ are present in the same electrode, labeled as **BVO|SiO|Ru**_**Woc**_ **-Ru**_**Med**_. The SABs design based on noncovalent alkyl–alkyl interactions, significantly ensures *a* robust hybrid structure of the molecular complexes with the **BVO|SiO**. This architecture generated a thin hydrophobic layer between the BiVO_4_ absorber and the molecular complexes, enhancing its overall stability without precluding efficient charge transfer between the Ru complexes and the BiVO_4_ absorber. Under 1 sun illumination at pH 7, the **BVO|SiO|Ru**_**Woc**_ **-Ru**_**Med**_ hybrid photoelectrode achieved steady photocurrent densities of approximately 2 mA/cm^2^ for about 1 h at an applied potential of *E*_app_ = 0.82 V vs NHE (1.23 vs RHE), with a 98% Faradaic efficiency for water oxidation to O_2_. This corresponds to a turnover number greater than 23,500 per metal center, or 280,000 per Ru oligomer. The photoelectrochemical performance obtained for **BVO|SiO|Ru**_**Woc**_ **-Ru**_**Med**_, at neutral pH is comparable to or exceeds those of related **BiVO**_**4**_ photoanodes employing oxide-based water-oxidation catalysts yet the amount of catalyst needed was more than 2 orders of magnitude lower under the same conditions.

In summary, the SAB-based molecular hybrid material **BVO|SiO|Ru**_**Woc**_ **-Ru**_**Med**_ functions as a robust and efficient photoanode for water oxidation to dioxygen, enabled by the effective integration of the optical and electronic properties of **BVO** with the tunability and molecular precision of Ru complexes acting as water-oxidation catalysts and electron mediators. This work demonstrates how the rational integration of molecular catalysts with tailored material surfaces can unlock synergistic reactivity and fully exploit the complementary advantages of molecular and solid-state components.

## Supplementary Material



## Notes

Additional information regarding experimental details, spectroscopy, electrochemistry and microscopy (PDF)
