# Supplementary material for: Water Oxidation by Molecular Catalyst Bilayers Self-Assembled on Bismuth Vanadium Oxide (BVO) Photoelectrodes
Source: J Am Chem Soc. 2026 Aug 5;148(32):34604–14. doi: 10.1021/jacs.6c08907 (PMC13495748; doi:10.1021/jacs.6c08907)
Supplement: Supplementary file 1 [file ja6c08907_si_001.pdf]

Supporting information for:

Water Oxidation by Molecular Catalyst Bilayers Self-Assembled on Bismuth Vanadium Oxide (BVO) Photoelectrodes

Paula Tris-Marzo,[a] Pierpaolo Vecchi,[b] Marcos Gil-Sepulcre*,[c] Gerald J. Meyer*,[b] and Antoni Llobet*,[a]

[a] Institute of Chemical Research of Catalonia (ICIQ), Av. Països Catalans 16, Tarragona, 43007, Spain.

[b] Department of Chemistry, University of North Carolina at Chapel Hill, Chapel Hill, NC 27599, USA

[c] Departament de Química Orgànica, Facultat de Química, Universitat de València, Av. Vicent Andrés Estellés 19, 46100 Burjassot, Spain

email corresponding authors: marcos.gil@uv.es, gjmeyer@unc.edu and allobet@iciq.cat

Experimental Procedures 3

Materials 3

Instrumentation 3

Photoelectrochemical measurements 5

Oxygen measurements 5

Incident Photon-to-Current Efficiency (IPCE) measurements 6

Preparation of hybrid photoanodes 6

Preparation of BVO photoanodes 6

Preparation of BVO|SiO 7

Preparation of BVO|SiO|Ru_{Woc} 7

Preparation of BVO|SiO|Ru_{Med} 8

Preparation of BVO|SiO|Ru_{Woc}-Ru_{Med} 8

Contact angle 11

Transient absorption spectroscopy 13

Photoelectrochemical measurements 18

References 23

Experimental Procedures

Materials

All chemicals used in this work were obtained from Sigma Aldrich and have been used without further purification unless explicitly indicated. Ru-complexes Ru_{Woc} and Ru_{Med} were prepared following the procedures reported in the literature.[1] High purity de-ionized water used for the electrochemistry experiments was obtained by passing distilled water through a nanopure Mili-Q water purification system. Fluorine-doped tin oxide (FTO) TEC 15 glass was purchased from Xop Física with a surface resistivity 12-14 Ω/sq 15 A resistance and 2.1 mm of thickness was used for electrochemical measurements.

Instrumentation

pH meter: a CRISON, Basic 20⁺ was used for determining the pH of the solutions, calibrated before measurements throughstandard solutions at pH 2.0, 4.0, 7.0, 9.21 and 11.0.

Tip sonicator: a Fisher Scientific model 120 sonicator served to prepare homogeneous solutions that were employed for the construction of the photoanodes.

Spin coater: a Laurell WS 650 spin coater was employed for the construction of the photoanodes.

Muffle furnace: a HD-230-PAD P muffle furnace from Hobersal was used for the intermediate and final annealing required to prepare the photoanodes.

UV-vis spectrometry: a Cary 50 (Varian) UV-vis spectrophotometer was used to carry out the UV-vis spectroscopy.

Resonance Raman Spectroscopy: spectra were measured with a Renishaw in Via Reflex RAMAN confocal microscope instrument equipped with an Ar ion laser, operating at 514 nm.

ICP–MS Spectrometry**:** an Agilent 7900 inductively coupled plasma mass spectrometer, equipped with an SPS 4 autosampler with a capacity of up to 240 positions, was used for elemental analysis. Samples were digested using a mixture of HNO_3 and HCl. Two digestion replicates were prepared for the samples. The digested solutions were diluted in 1% (v/v) HCl prior to injection.

Transient Absorption Spectroscopy: transient absorption spectroscopy with nanosecond time resolution was performed in a standard three electrode quartz cell. The electrode (2x1 cm) was placed on a teflon holder at 45° of both the laser and arc-lamp, Ag/AgCl (KCl 3M) was used as a reference electrode while a Pt mesh served as the counter electrode. Pulsed excitation was done using a RADIANT X30 tunable laser system: Q-switched, pulsed (10Hz) Nd:YAG laser (Quantel laser By Lumibird, Qsmart 450mJ) tripled to 355 nm. The laser fluence at the samples was 2 mJ/cm^2 per pulse (λ= 355 nm) at 1 Hz. A 150 W xenon arc lamp aligned perpendicular to the laser was employed as the probe beam. To minimize photomultiplier tube (PMT) fatigue and sample degradation, two shutters positioned between the arc lamp/laser and the sample were operated with 30 ms opening intervals. The probe beam was focused onto the sample, subsequently collimated and directed into an Oriel Cornerstone 260 monochromator coupled to a Hamamatsu R928 PMT. Transient signals were acquired using a Teledyne LeCroy WaveSurfer 4024HD digital oscilloscope (200 MHz), equipped with variable bandwidth filters to enhance the signal-to-noise ratio. Timing and synchronization of the laser flashlamps, Q-switch, shutters and oscilloscope triggering were controlled by a Berkeley Nucleonics Corp. Model 577 digital delay generator. The overall temporal

response of the setup was approximately 15 ns. Single-wavelength kinetic traces were obtained by averaging 90–120 laser pulses to ensure sufficient signal-to-noise ratios.

Intensity Modulated Photocurrent Spectroscopy employed a WaveSurfer 4024HD, 200 MHz, 5 GS/s oscilloscope together with a Gamry Interface 1010B potentiostat, a DS345 30 MHz synthesized function generator and a DC2200 LED Driver with Pulse Modulation. The light source was an LED with a nominal peak wavelength of 365 nm that was sinusoidally modulated from 1 Hz to 1 kHz.

Scanning Electron Microscopy: a Thermo Fisher Scientific FESEM (Field Emission Scanning Electron Microscope), model Scios2, was used to capture the images. The imaging conditions for each sample are provided in the data bar of the image. WD represents the working distance, Det indicates the type of detector (T2 secondary electron detector was used in this case), HV denotes the electron acceleration voltage (set at 5.00 kV), and the electron beam current applied was 50 pA.

Atomic Force Microscopy: the Agilent 5500 AFM was utilized for atomic force microscopy. Measurements were conducted using a high-resolution supersharp tip with the following specifications: a height of 17 µm (range: 15 - 19 µm), a setback of 15 µm (range: 10 - 20 µm), and a tip radius of 1 nm.

Contact angle measurements: Holmarc's contact angle meter (Model No: HO-ED-M-01) was used for measuring contact angles of liquids over solid substrates. It has manual dispensing unit with camera interface for measuring contact angle of liquids, in this case, Milli-Q® water on solid surfaces. The surface over which contact angle was measured was held in horizontal position to find out contact angle using sessile drop method.

Solar simulator: Newport 300 W ozone-free Xe arc lamp solar simulator, equipped with water and AM 1.5G air mass filters, was used for simulated sunlight irradiation. The light intensity was calibrated to 1 sun (100 mW/cm^2) using a silicon photodiode (Newport power meter model 919-P).

Incident Photon-to-Current Efficiency (IPCE) measurements: the IPCE system was formed by an Oriel 150 W xenon lamp, a VIS-IR motorized monochromator, a light power meter and a calibrated solar cell.

Clark electrode: an OX-NPLR Clark-type oxygen needle sensor connected to a Unisense controller, with the signal recorded using a CH Instruments 730D potentiostat.

X-ray photoelectron spectroscopy (XPS) analysis was performed at SRCiT (URV) using a ProvenX-NAP (Specs Surface Nano Analysis GmbH, Berlin, Germany) spectrometer with a dual (AlKα = 1487 eV / AgLα = 2984 eV) monochromatic X-ray source (µFocus450). The measurements were collected under these conditions: power of X-ray of 100 W (AlKα anode), the hemispherical analyzer operating in the main fixed analyzer transmission mode, entrance slit of 7x20mm and exit slit open with mesh. The data were recorded with a PHOIBOS 150 NAP 1D-DLD and the total pressure in the main vacuum chamber during analysis was better than 1·10-8 mbar. Survey spectrum was collected at 80 eV of pass energy and 0.7 eV of step size. To obtain more detailed information about chemical environment and oxidation states, high-resolution spectra were recorded from individual peaks at 30 eV of pass energy, 0.3 Dwell Time and 0.05 eV of step. Due to the electrical properties of the samples, a flood gun source (COSCON - FG 22/35, SPECS) had to be used to compensate for charging problems. Data processing was performed using CasaXPS processing software version 2.3.26.PR1.0 (Casa Software Ltd., Teignmouth, UK). The atomic concentrations of the detected elements were calculated using integral peak intensities and the sensitivity

supplied by the manufacturer. Binding energies were referenced to the C1s peak at 284.8 eV (adventitious carbon).

Photoelectrochemical measurements

All photoelectrochemical experiments were conducted using an IJ-Cambria CHI-730D potentiostat in a single-compartment, three-electrode Teflon cell for linear sweep voltammetry (LSV) and chopped controlled potential electrolysis (chopped-CPE) and controlled potential electrolysis (CPE). A BVO (2 × 1 cm) electrode served as the working electrode (WE), with electrical contact established via the FTO layer. A platinum coil was used as the counter electrode (CE), and a Hg/Hg_2SO_4 (K_2SO_4 sat.) electrode functioned as the reference electrode (RE). All potentials reported were converted to the normal hydrogen electrode (NHE) scale using the following equation:

$$V_{NHE} = V_{Hg/HgSO_4} + 0.645 \quad (1)$$

LSV and Chopped-CPE experiments were conducted in a 50 mL Teflon cell with a controlled exposed area of 0.5 cm^2 under front-side illumination and a scan rate was 10 mV/s, unless stated otherwise. Detailed experimental conditions, including the solution composition, applied potential, and duration of each experiment, are detailed in the corresponding figure captions. For Chopped-CPE and CPE experiments, photocurrents were recorded at a bias of 0.817 V vs. NHE using illumination from a solar simulator. The light intensity was calibrated to 1 sun (100 mW/cm^2) using a silicon photodiode (Newport power meter model 919-P). All the experiments were performed under 1 sun illumination. All electrochemical data presented in this work were IR-compensated, except for CPE experiments. The cell resistance typically ranged from 80 to 120 Ω, with an IR compensation factor of 85%.

Photoelectrochemical measurements were performed in aqueous media using a phosphate buffer (NaPi) with an ionic strength μ = 1 M (pH 7) to maintain a stable pH and provide ionic conductivity. Preparation was made using Mili-Q water to a final volume of 2 L, with the following components: NaH_2PO_4 (0.305 mol, 36.630 g) and Na_2HPO_4 (0.565 mol, 80.173 g). The pH was adjusted to 7 by adding small amounts of 1 M NaOH or 1 M HCl as needed.

Oxygen measurements

To quantify the produced oxygen, a CPE was performed to evaluate the oxidation of water to dioxygen using the prepared BVO photoelectroanodes. Experiments were carried out in NaPi at pH 7 with an applied potential (E_{app}) of 0.817 V vs. NHE in a two-compartment quartz cell, where the counter electrode (CE) was separated by a frit from the working electrode (WE) and reference electrode (RE). The setup included a WE, RE and CE, along with a magnetic stirring bar. The cell was sealed with a septum The cell was sealed with a septum to prevent gas leakage. BVO-based photoanodes (S = 2 cm^2) served as the WE, with the electrical connection established via the fluorine-doped tin oxide (FTO) substrate. A $Hg/HgSO_4$ (K_2SO_4 sat.) electrode was used as the RE, and a platinum coil was employed as the CE. The cell was filled with 45 mL of a pH 7 NaPi solution. Oxygen evolution during the experiments was monitored using a Clark electrode positioned in the working electrode compartment to detect gaseous oxygen in the headspace. After each experiment, the oxygen sensor was calibrated by performing several injections of 100 µL of air (O_2 = 21%) into the cell using a gas-tight Hamilton syringe.

Faradaic efficiency (FE) was calculated by correlating the total charge passed during the CPE with the amount of detected oxygen, assuming a four-electron transfer mechanism for water oxidation.

$$\mathrm{FE}\ (\%) = \frac{\mathrm{mol\ O_2}}{\left(\frac{\mathrm{Q}}{\mathrm{F\ n_{e^-}}}\right)} \cdot 100 \qquad (2)$$

Where mol O_2 denotes the amount of oxygen detected using the Clark-type electrode, Q represents the charge passed during the CPE experiment, F is the Faraday constant (96,485 C/mol), and n_{e^-} is the number of electrons involved in the oxidation process (4 for water oxidation).

Incident Photon-to-Current Efficiency (IPCE) measurements

IPCE was measured using a system composed of an Oriel 150 W xenon lamp, a VIS-IR motorized monochromator, a Gentec light power meter and a Keithley multimeter. Calibration was performed by measuring a calibrated solar cell under the fiber optic light path, verifying that the peak response at ~560 nm reached 43–45%; if necessary, the distance between the fiber and the sample was adjusted. Once calibrated, the measurements were conducted using a single-compartment Teflon cell, where the exposed BVO working electrode area was 0.5 cm². Measurements were performed under front-side illumination, with a Hg/$HgSO_4$ (K_2SO_4 sat.) as RE and a Pt coil as CE. A phosphate buffer solution (NaPi) with an ionic strength μ = 1.0 M ionic strength and pH 7 was used as electrolyte, and a bias voltage of 0.817 V vs. NHE was applied during the measurements. IPCE values were calculated using the following equation:

$$\mathrm{IPCE}\ (\%) = \frac{1240 \cdot \mathrm{J_{ph}}}{\lambda \cdot \mathrm{P_{light}}} \cdot 100 \qquad (3)$$

Where J_{ph} is the photocurrent density (A·cm^{-2}), λ refers to the wavelength (nm) and P_{light} is the incident light power density (W·cm^{-2})

Preparation of hybrid photoanodes

Preparation of BVO photoanodes

The BVO electrodes, which are in fact FTO/SnO_2/WO_3/$BiVO_4$ electrodes, were fabricated by sequentially spin-coating different layers of each material following an established procedure reported in the literature, with minor modifications.[2] For each coating layer, 100 µL of the respective solution was deposited onto a 2x2 cm² electrode using a spin coater. The spin coating process consisted of two steps: the first step involved a ramp acceleration to 2500 rpm for two seconds, and the second step maintained the speed at 2500 rpm for 40 seconds. All ultrasonication steps were performed using a tip horn sonicator set to 70% amplitude, with a pulse cycle of 30 seconds on and 10 seconds off. The layer deposition process is outlined in the following scheme:

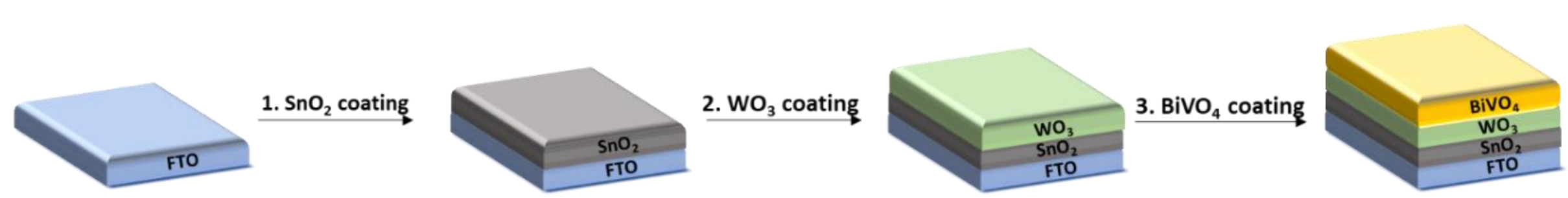

Figure S1. Schematic representation of the preparation of BVO electrodes.

FTO preparation. The slides are placed in a beaker containing a solution of Hellmanex detergent (1:100) in Milli-Q water and subjected to an ultrasonic bath for 10 minutes, followed by rinsing with Milli-Q water. Next, the beaker is refilled with fresh Milli-Q water, and the slides undergo another 10 minutes of

ultrasonic treatment, then rinsed with ethanol. A third ultrasonic cleaning session is performed for 10 minutes with pure ethanol, followed by rinsing with clean ethanol. Finally, the slides are dried using Kimtech™ optical paper. The dried slides are then heated for 10 minutes at 100 °C in an oven and subsequently for 30 minutes at 495 °C in a muffle furnace. Following this preparatory phase, the FTO plates were cut into segments of 2x2 cm^2 using a diamond glass cutter.

1. SnO_2 coating. The SnO_2 coating solution was prepared by dissolving 0.701 g of tin(IV) chloride pentahydrate and 1 g of polyethylene glycol (M.W. = 6000) in 10 mL of 2-methoxyethanol. The mixture was ultrasonicated for 20 minutes using a tip horn sonicator. The coating was applied in 9 layers, with each layer being followed by an intermediate annealing step in a muffle furnace at 350 °C for 5 minutes. After the final layer was applied, the sample underwent a final annealing at 450 °C for 1 hour.

2. WO_3 coating. To prepare the WO_3 coating solution, 1.25 g of tungstic acid was dissolved in 28 mL of 30% hydrogen peroxide solution. The solution was heated on a hot plate at 150 °C until its volume was reduced to 3.5 mL. Then, 31.5 mL of 2-methoxyethanol was added, and the solution was ultrasonicated for 1 hour. The coating was applied in 5 layers, with intermediate annealing after each layer at 350 °C for 5 minutes. The final annealing was conducted at 500 °C for 1 hour.

3. $BiVO_4$ coating. The $BiVO_4$ coating solution was prepared by dissolving 0.404 g of bismuth(III) nitrate pentahydrate and 0.221 g of vanadyl acetylacetonate in a solvent mixture of acetic acid, 2-methoxyethanol, and acetylacetone (volume ratio 1:4:1). The solution was stirred magnetically for 30 minutes and ultrasonicated for 30 minutes using a tip horn sonicator. This coating was applied in 7 layers, with intermediate annealing at 350 °C for 5 minutes after each layer. The final annealing was performed at 500 °C for 2 hours obtaining the BVO/WO_3/SnO_2/FTO electrodes as illustrated in Figure S1.

Preparation of BVO|SiO

As described in Figure S2, the BVO electrode was first immersed in a 0.1 M solution of OTMSi in DCM for 30 minutes removed from the solution and rinsed with rinsed with DCM, air-dried, to generate **BVO|SiO**.

Preparation of BVO|SiO|Ru_{Woc}

The BVO|SiO electrode was introduced into a 2 mM solution of Ru_{Woc} in a 85:15 (v/v) TFE/DCM mixture overnight. Finally, a rinse with a 97:3 (v/v) TFE/DCM mixture was performed, followed by air-drying to generate **BVO|SiO|Ru_{Woc}**.

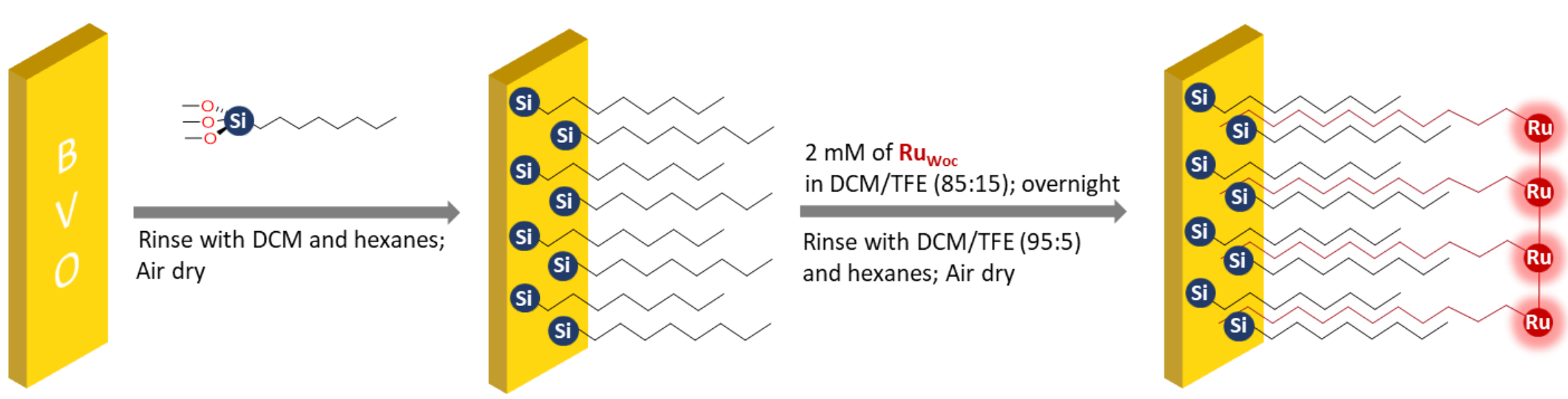

Figure S2. Schematic representation of the preparation of **BVO|SiO|Ru_{Woc}**.

Preparation of BVO|SiO|Ru_{Med}

The BVO|SiO electrode was introduced into a 0.1 M solution of Ru_{Med} in DCM for 2 hours. This generates the electrode **BVO|SiO|Ru_{Med}**, that was rinsed with DCM and hexane, followed by air-drying.

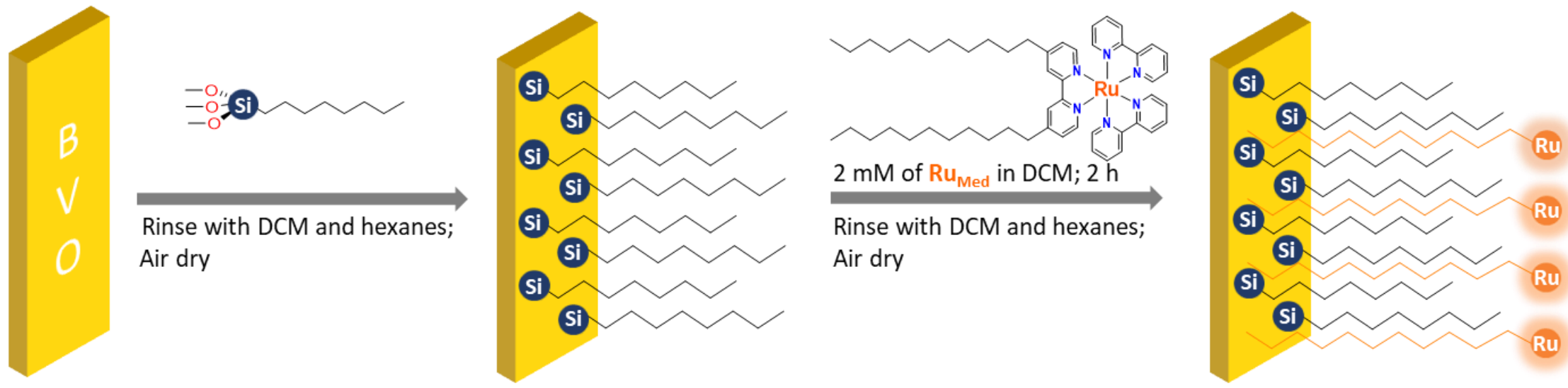

Figure S3. Schematic representation of the preparation of **BVO|SiO|Ru_{Med}**.

Preparation of BVO|SiO|Ru_{Woc}-Ru_{Med}

The BVO|SiO electrode was placed into a 2 mM solution of Ru_{Woc} in an 85:15 (v/v) of DCM/TFE mixture overnight. The electrode was then rinsed with a 97:3 (v/v) DCM/TFE mixture, hexanes and air-dried. Following this, the electrode was soaked in a 2 mM solution of Ru_{Med} in dichloromethane for 2 hours. This generates the **BVO|SiO|Ru_{Woc}-Ru_{Med}** electrode, that was rinsed with DCM and hexanes and then air-dried.

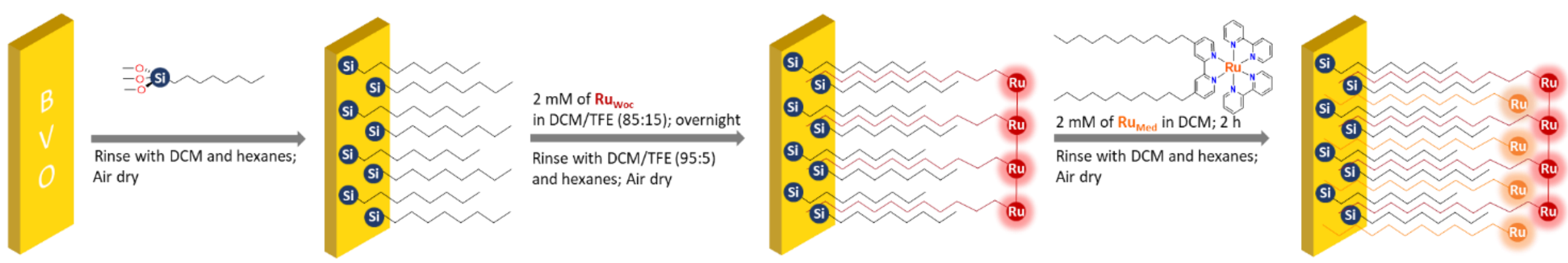

Figure S4. Schematic representation of the preparation of **BVO|SiO|Ru_{Woc}-Ru_{Med}**.

Microscopy

(a) (b) (c)

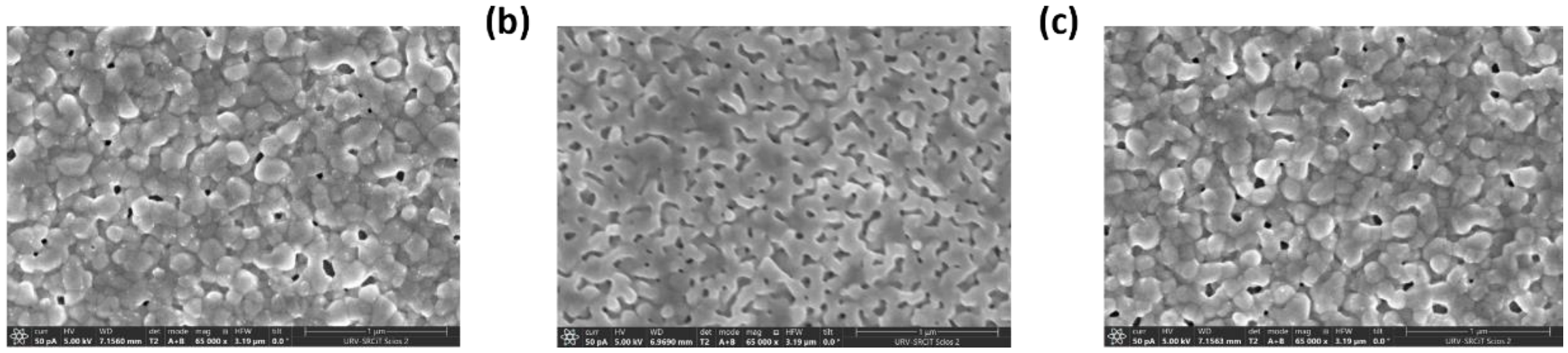

Figure S5. Field Emission Scanning Electron Microscopy (FESEM) images of (a) **BVO**; (b) **BVO|SiO**; (c) **BVO|SiO|Ru_{Woc}**.

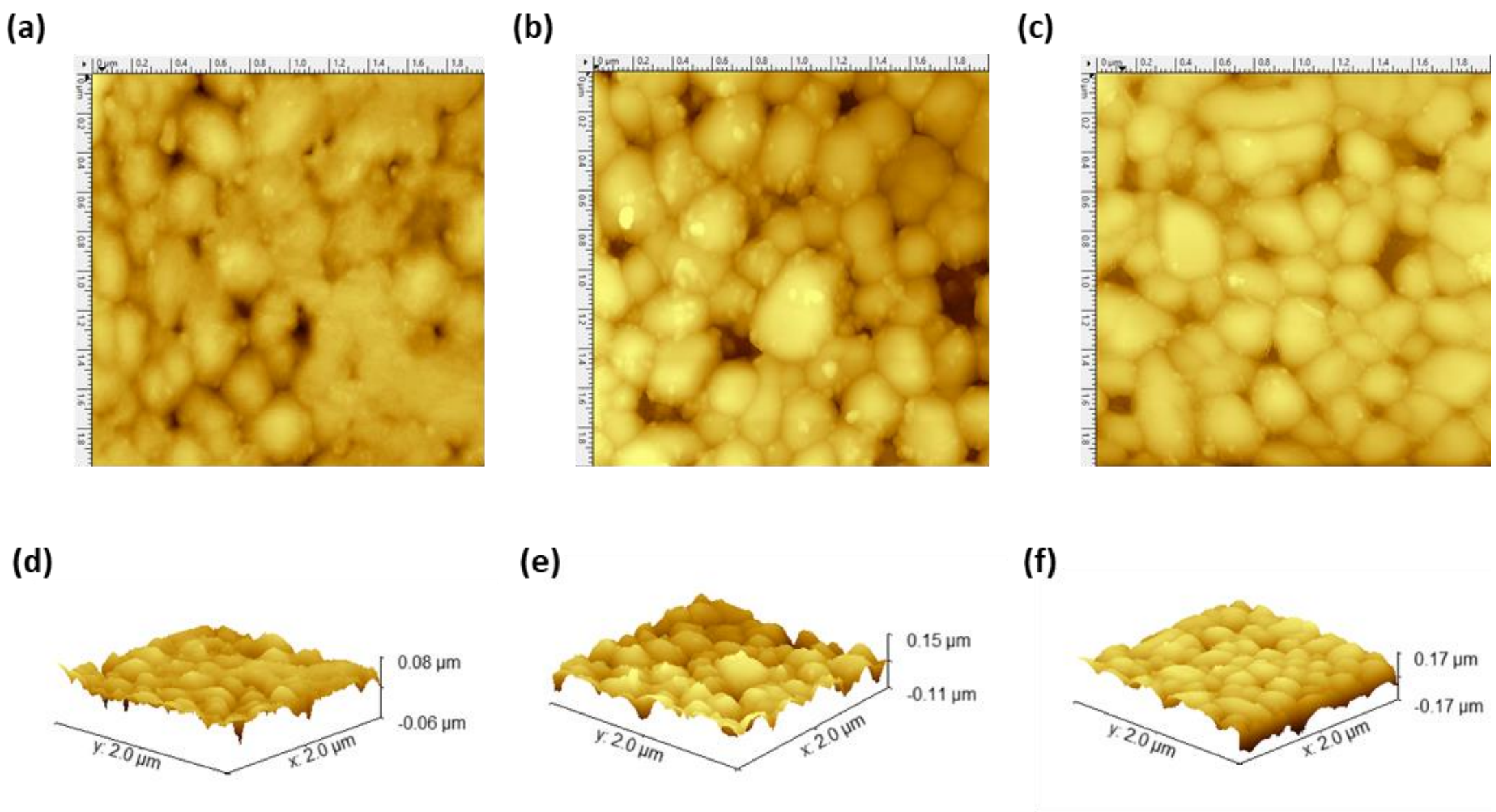

Figure S6. Atomic Force Microscopy (AFM) images of (a) **BVO**; (b) **BVO|SiO**; (c) **BVO|SiO|Ru$_{Woc}$**. AFM three-dimensional image of (d) **BVO**; (e) **BVO|SiO**; (f) **BVO|SiO|Ru$_{Woc}$**.

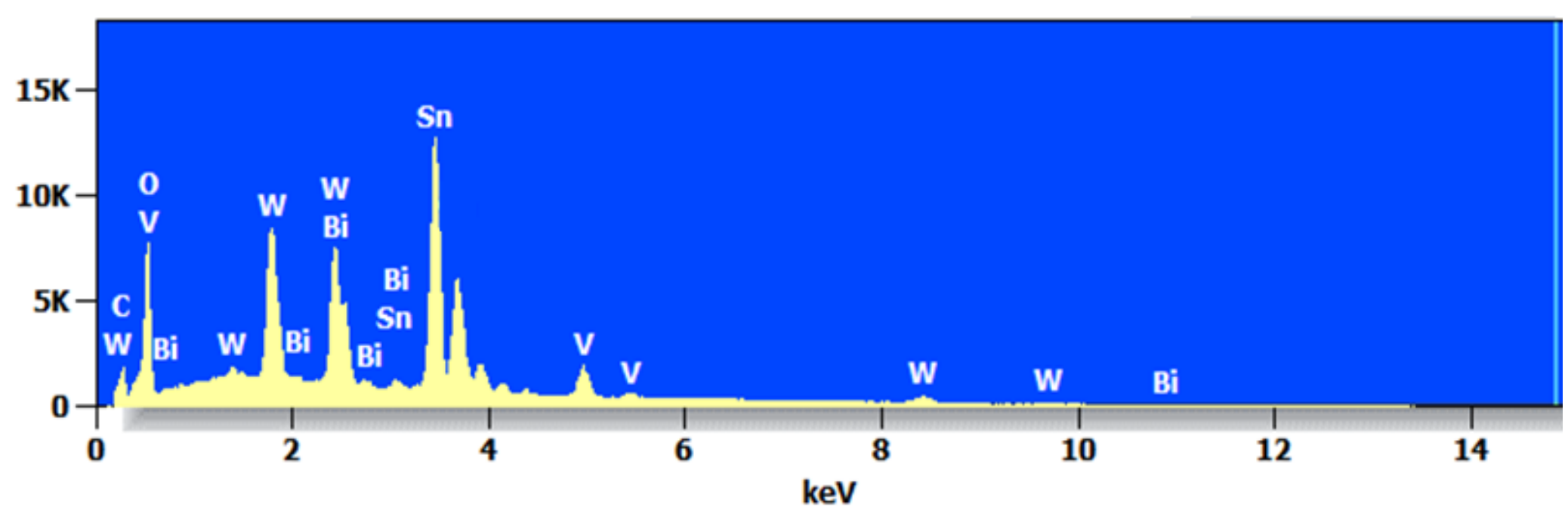

Figure S7. Energy dispersive X-ray spectroscopy (EDX) spectra of **BVO|SiO|Ru$_{Woc}$**.

Table S1. Quantitative results of EDX spectroscopy of **BVO|SiO|Ru$_{\mathbf{WOC}}$** in Figure S7.[a]

Element	Weight (%)	Weight error (%)	Atom (%)
**Bi**	18.16	± 0.14	5.27
**Sn**	45.62	± 0.28	23.33
**W**	16.38	± 0.12	5.41
**V**	4.22	± 0.05	5.02
**C**	1.31	± 0.02	6.68
**O**	14.30	± 0.46	54.27
**Total**	100.00	-	100.00

[a] Ru is not detected since its concentration is below the instrument's detection limit.

Contact angle

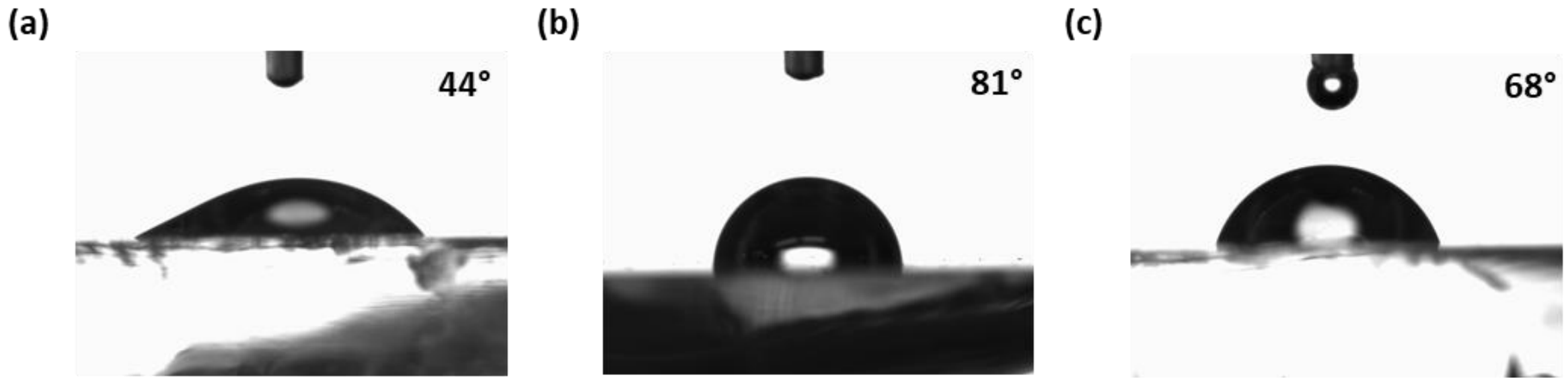

Figure S8. Contact angles of: (a) **BVO**; (b) **BVO|SiO**; (c) **BVO|SiO|Ru_{Woc}**.

UV-vis spectroscopy

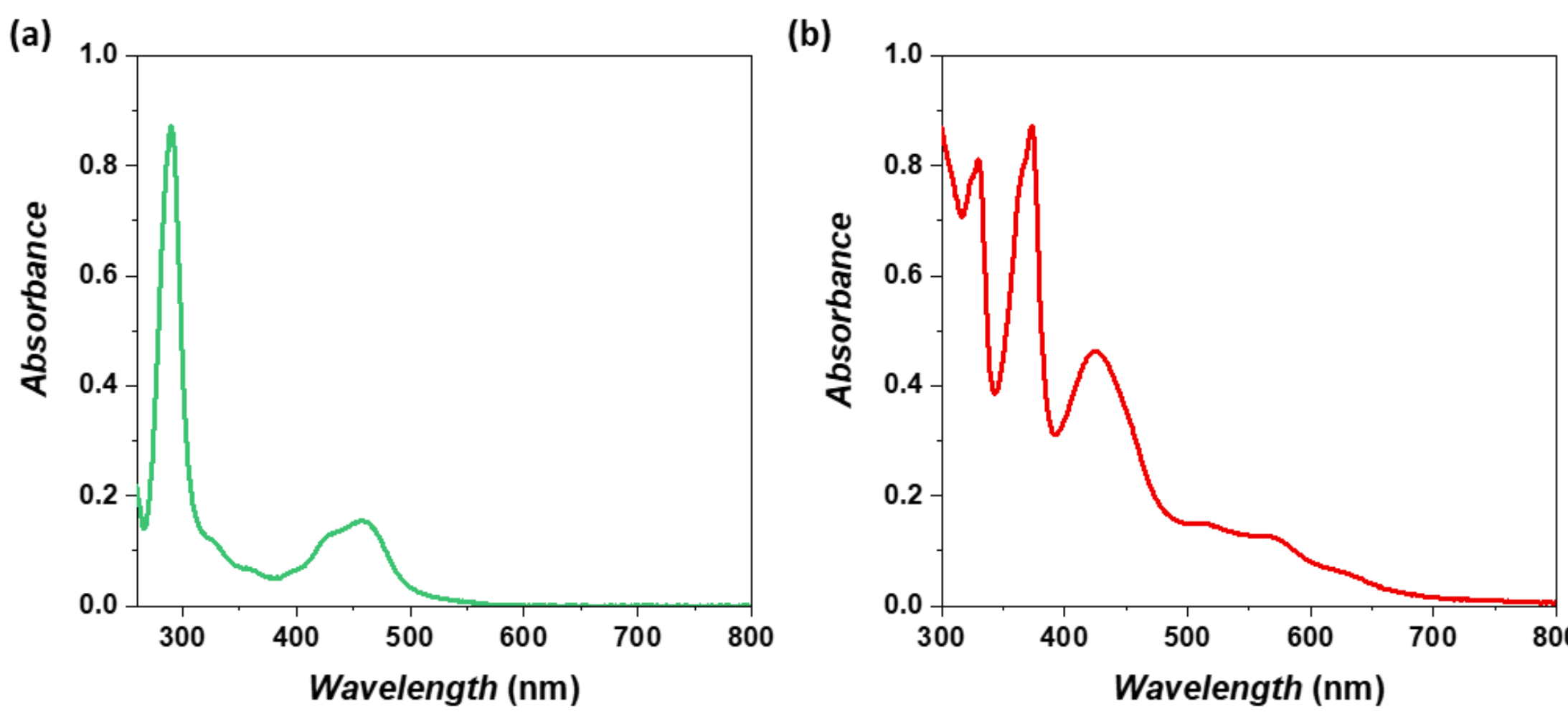

Figure S9. UV-vis absorption spectrum of: (a) 0.02 mM solution of **Ru_{Med}** in DCM; (b) 0.1 mM solution of **Ru_{Woc}** in TFE.

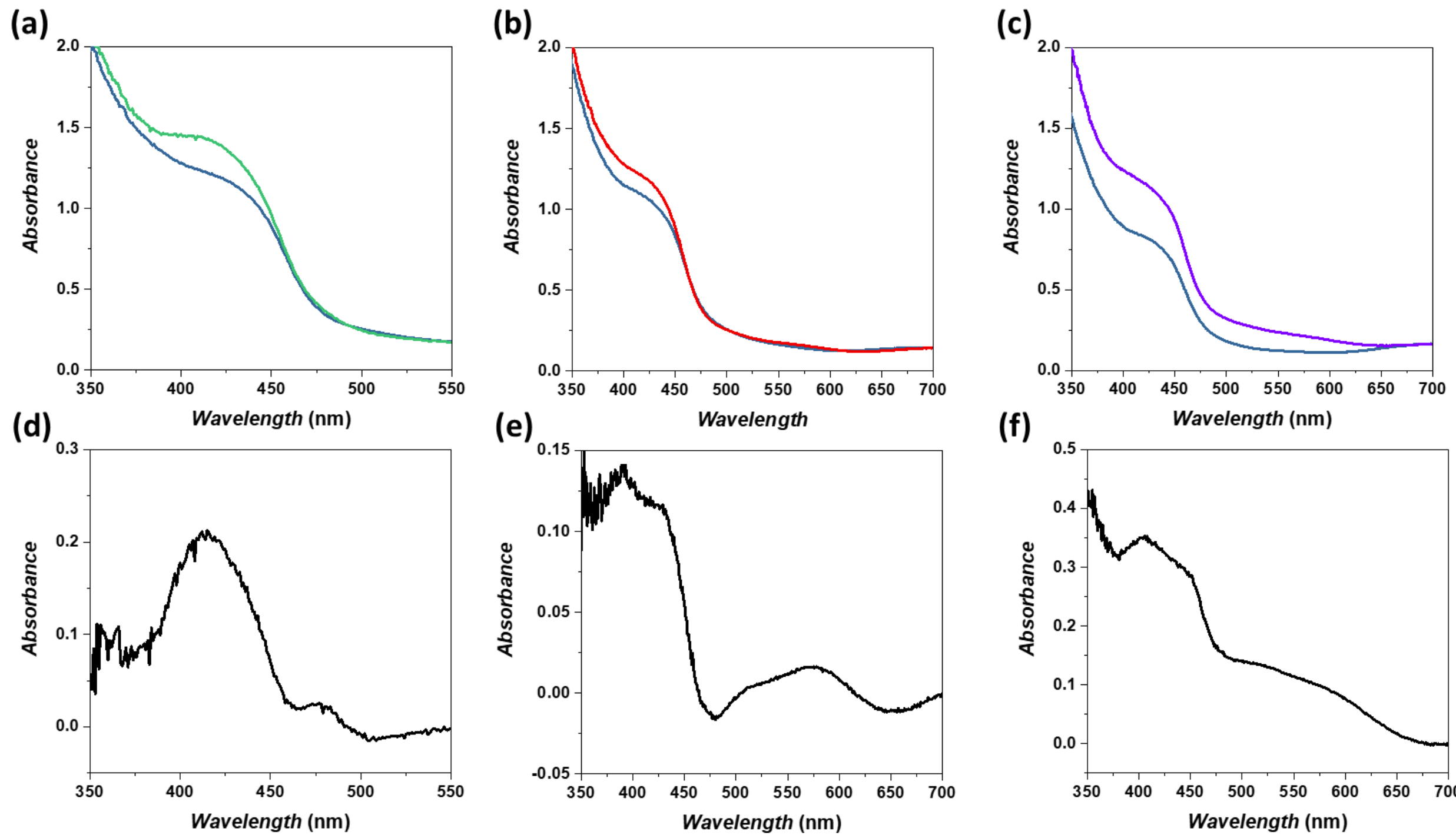

Figure S10a. Solid state UV-vis absorption spectra of (a) **BVO|SiO|Ru$_{Med}$** (green line) and **BVO|SiO** (blue line); (b) **BVO|SiO|Ru$_{Woc}$** (red line) and **BVO|SiO** (blue line); (c) **BVO|SiO|Ru$_{Woc}$-Ru$_{Med}$** (purple line) and **BVO|SiO** (blue line) and **BVO|SiO** subtracted spectra of: (d) **BVO|SiO|Ru$_{Med}$**; (b) **BVO|SiO|Ru$_{Woc}$**; (c) **BVO|SiO|Ru$_{Woc}$-Ru$_{Med}$**.

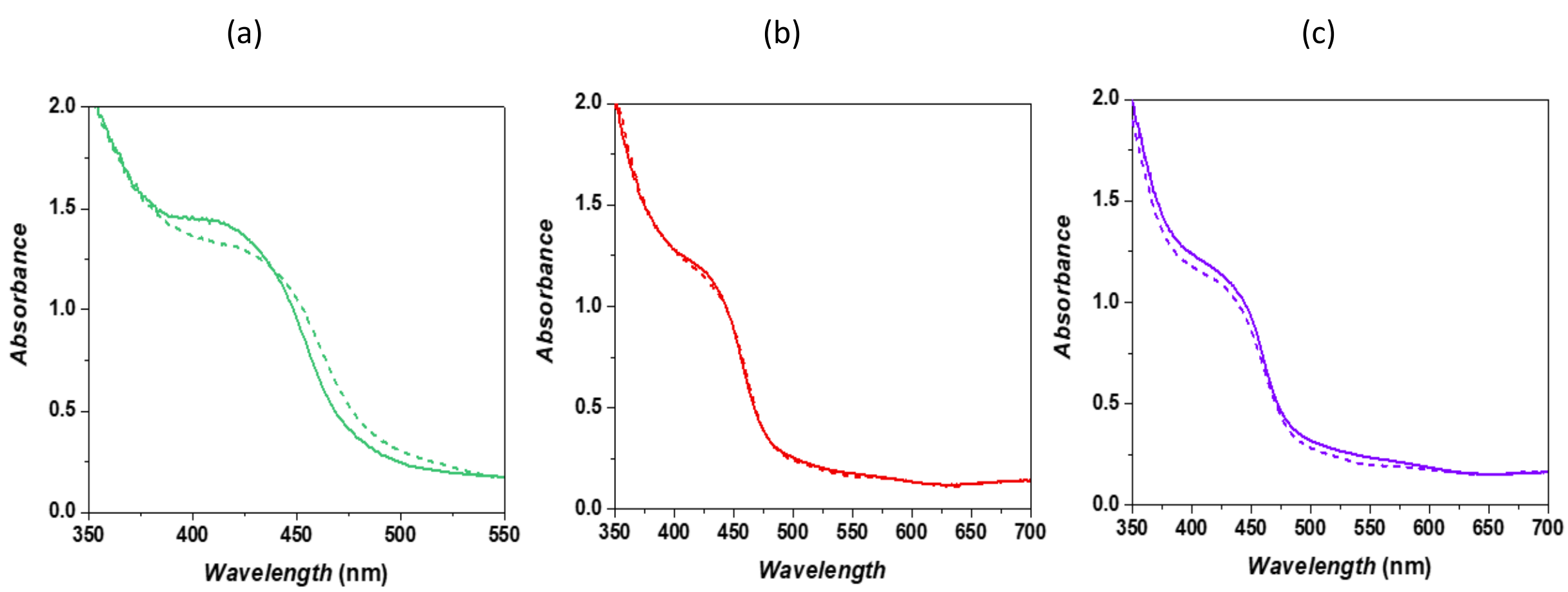

Figure S10b. Solid state UV-vis absorption spectra before (solid line) and after (dashed line) CPE in μ = 1.0 M pH 7 phbf aqueous solution under 1 sun illumination at E_{app} = 0.817 V vs. NHE for 1 hour of (a) **BVO|SiO|Ru$_{Med}$**; (b) **BVO|SiO|Ru$_{Woc}$**; and (c) **BVO|SiO|Ru$_{Woc}$-Ru$_{Med}$**.

Transient absorption spectroscopy

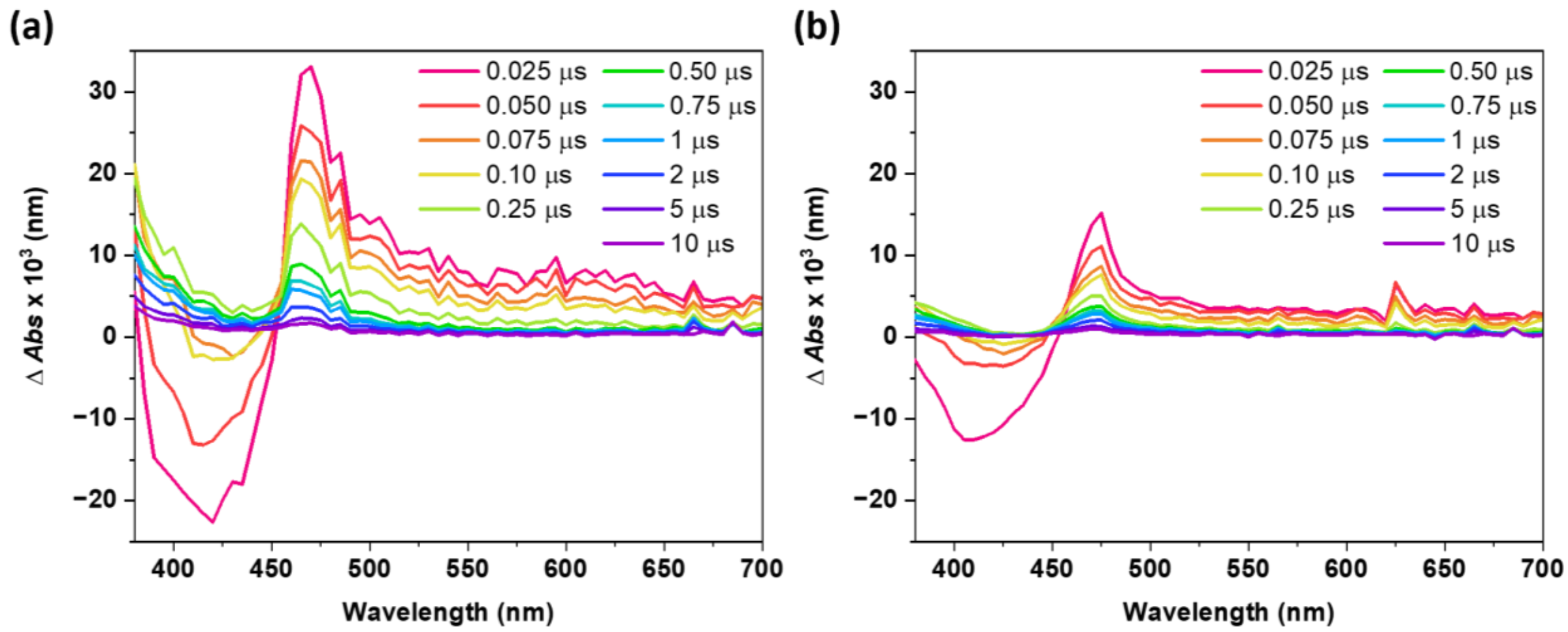

Figure S11. (a) Transient absorption difference spectra measured after pulsed 355 nm excitation (6 ns, 2 mJ/pulse, 1 cm^2 illuminated area) of BVO in absence and (b) upon addition of 0.5 M Na_2SO_3 at 0 V vs. NHE in in 1 M pH 7 NaPi aqueous solution, at the indicated time delays.

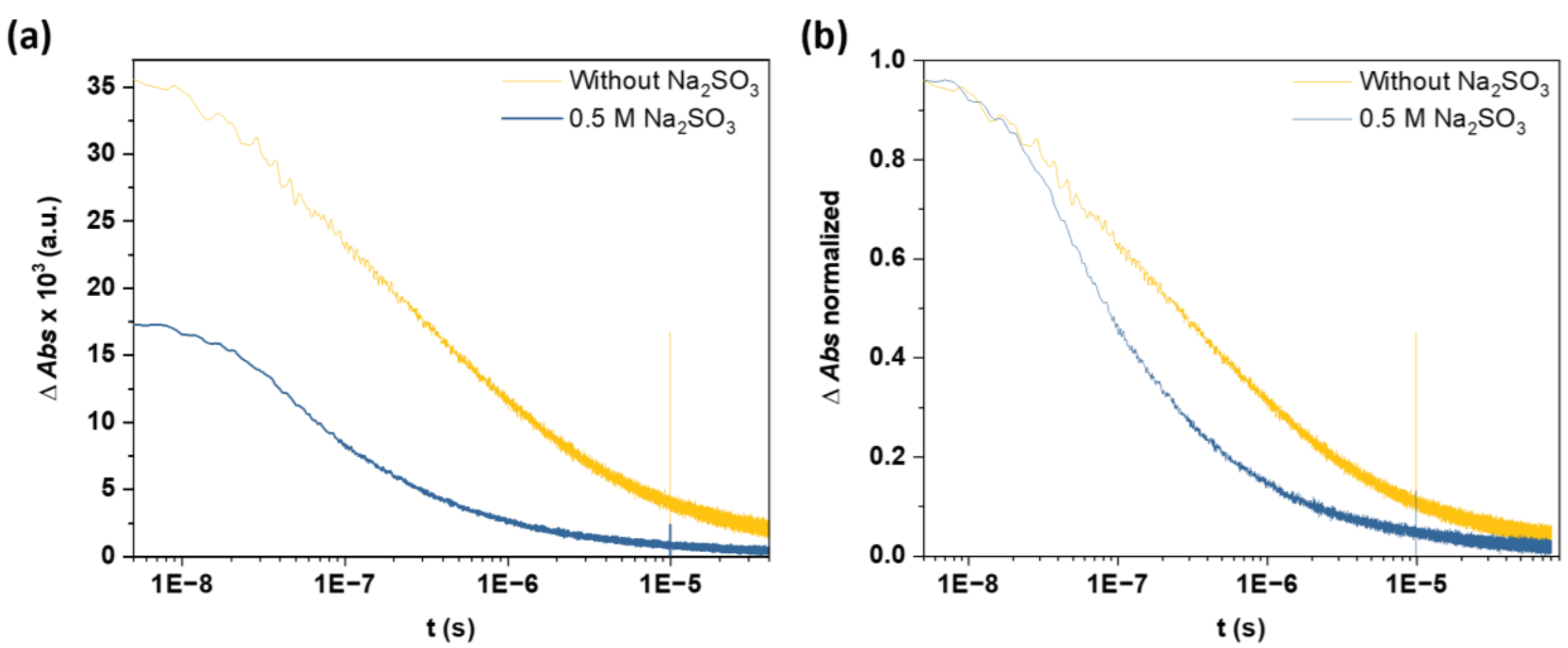

Figure S12. (a) Non- normalized and (b) normalized transient absorption difference spectra measured after pulsed 355 nm excitation (6 ns, 2 mJ/pulse, 1 cm^2 illuminated area) of BVO in absence (yellow trace) and upon addition (blue trace) of 0.5 M Na_2SO_3 at 0 V vs. NHE in in 1 M pH 7 NaPi aqueous solution.

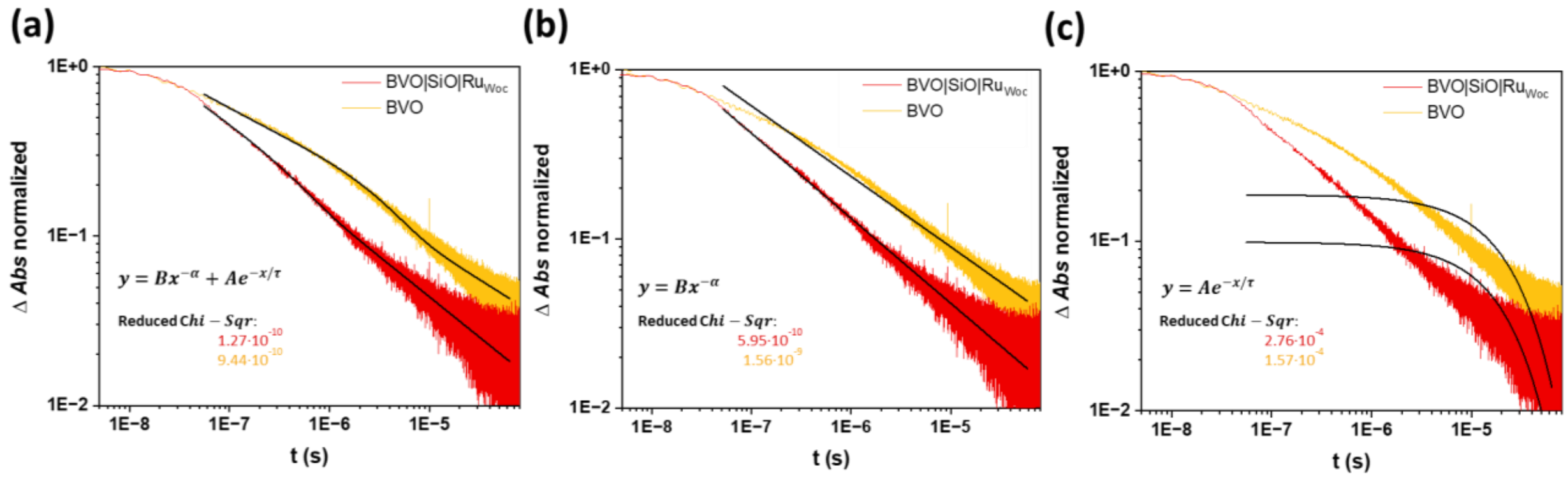

Figure S13. Comparison between different kinetic models overlaid on absorption changes of **BVO|SiO|Ru$_{Woc}$** (red trace) and **BVO** (yellow trace) monitored at 470 nm upon pulsed 355 nm excitation (6 ns, 2 mJ/pulse, 1 cm^2 illuminated area) at 0.82 V vs. NHE: (a) power law plus exponential, (b) double exponential, (c) triple exponential, (d) stretched exponential and (e) logarithmic. The corresponding mathematical expression for each fit is specified in the corresponding figure. The goodness of the fit is reported in the inset in terms of χ_v^2.

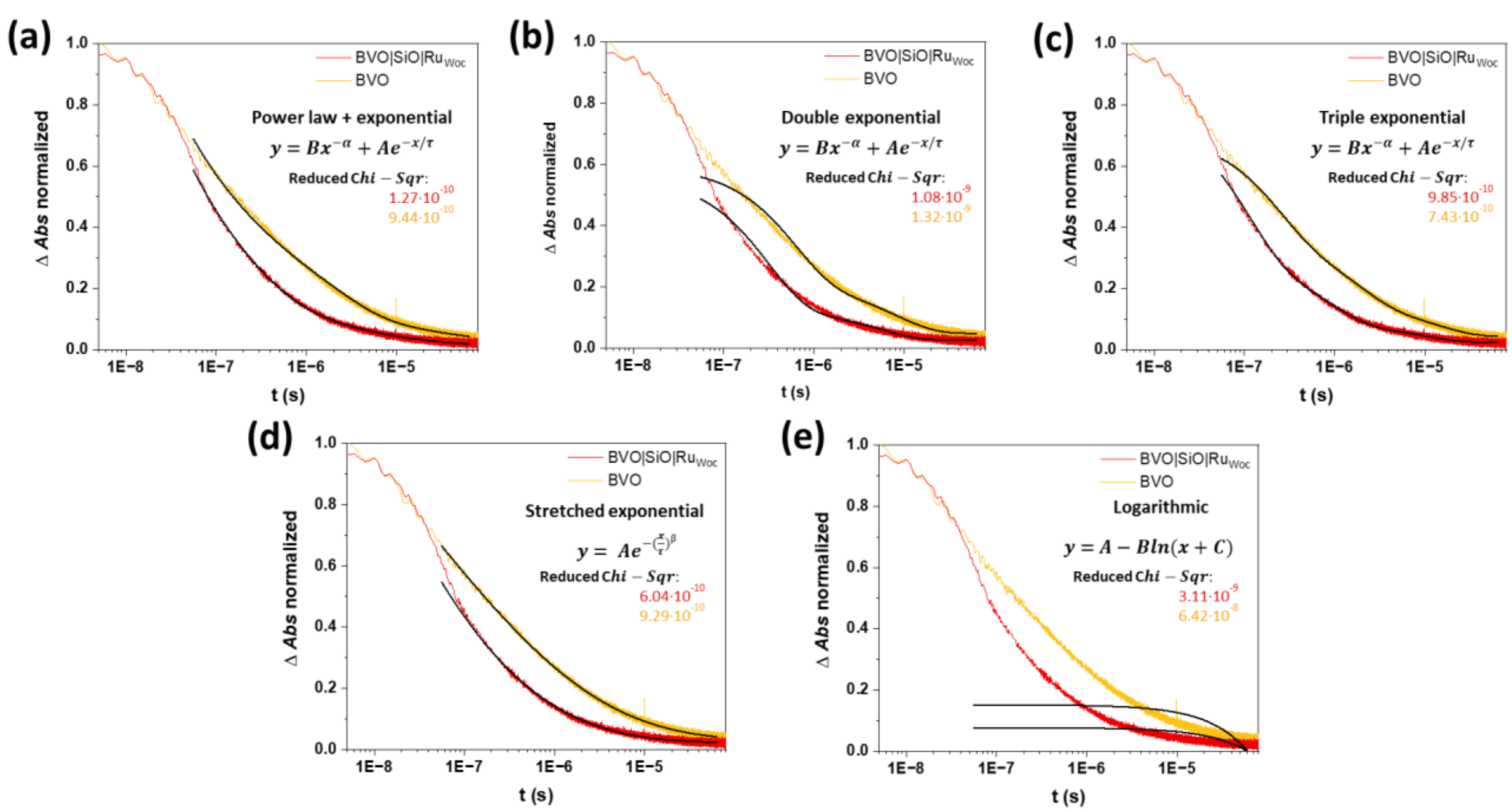

Figure S14. Analysis of the contribution of each component of the power law exponential fitting on absorption changes of **BVO|SiO|Ru$_{Woc}$** (red trace) and **BVO** (yellow trace) monitored at 470 nm upon pulsed 355 nm excitation (6 ns, 2 mJ/pulse, 1 cm^2 illuminated area) at 0.82 V vs. NHE: (a) power law plus exponential, (b) power law component and (c) exponential component. The corresponding mathematical expression for each fit is specified in the corresponding figure. The goodness of the fit is reported in the inset in terms of χ_v^2.

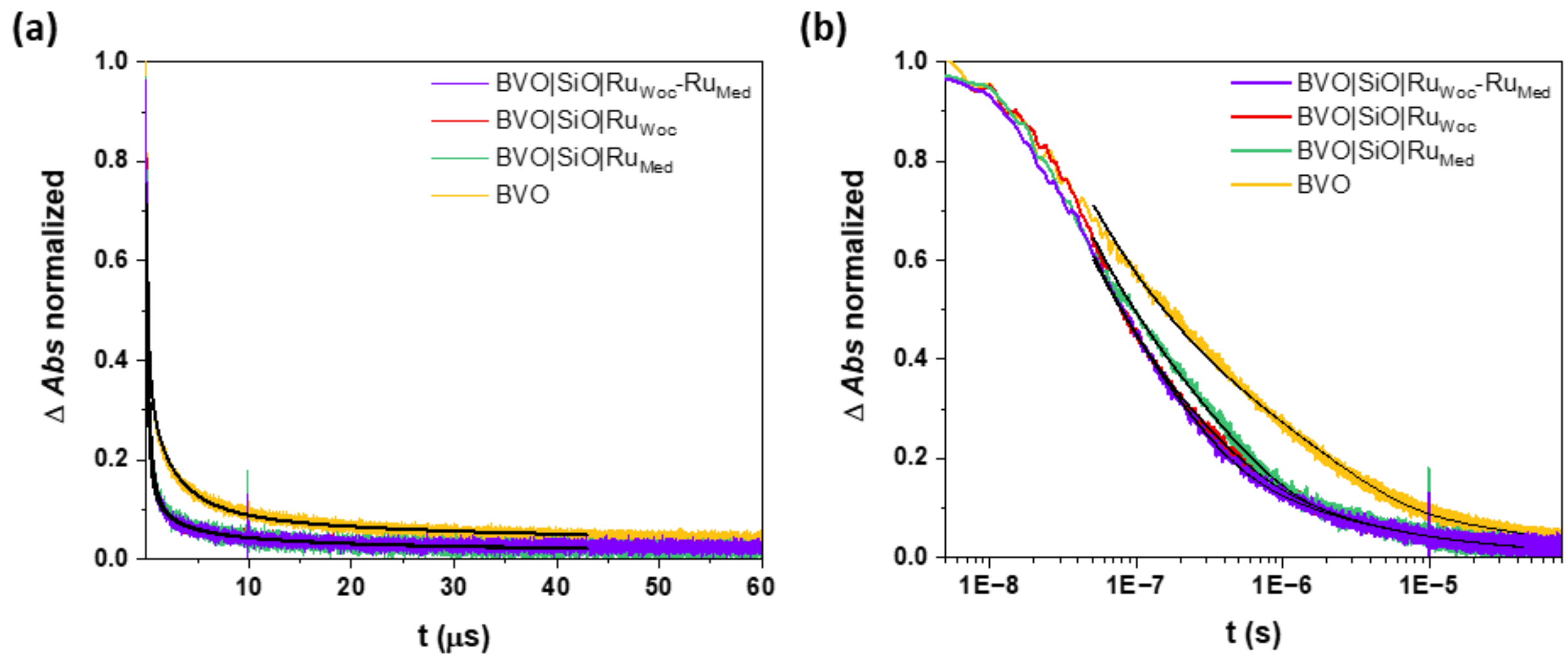

Table S15. (a) Absorption changes monitored at 470 nm upon pulsed 355 nm excitation (6 ns, 2 mJ/pulse, 1 cm^2 illuminated area) at 0.82 V vs. NHE for **BVO|SiO|Ru$_{Woc}$-Ru$_{Med}$** (purple trace), **Ru$_{Woc}$@OSi@BiVO$_4$/WO$_3$** (red trace), **BVO|SiO|Ru$_{Med}$** (green trace) and **BVO** (yellow trace). The kinetic data were normalized at 20 ns. (b) Spectra with represented exponential time scale.

Table S2. Kinetic parameters: B is the power law amplitude (s^{α}), exponent α, A is the exponential amplitude and τ is the time constant (s), obtained from power law plus exponential decay fitting monitored at 470 nm upon pulsed 355 nm excitation for the BVO photoanode at different applied potentials in a μ = 1 M pH 7 NaPi aqueous solution.

E_{app} (vs NHE)	B (s^{α})	A	A	τ (s)	R^2	Chi-Sq
**0 V**	$1.26\cdot10^{-3}$	0.41	$8.83\cdot10^{-2}$	$4.13\cdot10^{-6}$	0.98	$3.18\cdot10^{-9}$
**0.2 V**	$1.01\cdot10^{-3}$	0.40	$1.41\cdot10^{-2}$	$4.01\cdot10^{-6}$	0.98	$3.31\cdot10^{-9}$
**0.4 V**	$7.16\cdot10^{-4}$	0.40	$1.75\cdot10^{-1}$	$3.88\cdot10^{-6}$	0.98	$4.07\cdot10^{-9}$
**0.6 V**	$9.67\cdot10^{-4}$	0.38	$1.62\cdot10^{-1}$	$3.06\cdot10^{-6}$	0.98	$1.35\cdot10^{-9}$
**0.8 V**	$1.16\cdot10^{-3}$	0.37	$9.93\cdot10^{-2}$	$2.92\cdot10^{-6}$	0.98	$9.44\cdot10^{-10}$
**1 V**	$8.59\cdot10^{-4}$	0.37	$1.83\cdot10^{-1}$	$2.75\cdot10^{-6}$	0.98	$1.03\cdot10^{-9}$

Table S3. Kinetic parameters: B is the power law amplitude (s^{α}), exponent α, A is the exponential amplitude and τ is the time constant (s), A is the exponential amplitude obtained from power law plus exponential fitting monitored at 470 nm upon pulsed 355 nm excitation for the **BVO|SiO|Ru$_{Woc}$** photoanode at different applied potentials in a μ = 1 M pH 7 NaPi aqueous solution.

**E_{app} (vs NHE)**	**B (s^{α})**	**A**	**A**	**τ (s)**	**R^2**	**Chi-Sq**
**0 V**	$9.69{\cdot}10^{-5}$	0.52	$1.03{\cdot}10^{-3}$	$1.60{\cdot}10^{-6}$	0.99	$6.35{\cdot}10^{-10}$
**0.2 V**	$6.74{\cdot}10^{-5}$	0.55	$6.33{\cdot}10^{-2}$	$1.59{\cdot}10^{-6}$	0.99	$7.94{\cdot}10^{-10}$
**0.4 V**	$8.43{\cdot}10^{-5}$	0.54	$6.20{\cdot}10^{-1}$	$1.02{\cdot}10^{-6}$	0.98	$8.86{\cdot}10^{-10}$
**0.6 V**	$1.07{\cdot}10^{-4}$	0.52	$6.37{\cdot}10^{-2}$	$7.31{\cdot}10^{-7}$	0.99	$1.01{\cdot}10^{-9}$
**0.8 V**	$1.84{\cdot}10^{-4}$	0.48	$8.33{\cdot}10^{-2}$	$3.38{\cdot}10^{-7}$	0.99	$1.27{\cdot}10^{-10}$
**1 V**	$2.04{\cdot}10^{-4}$	0.46	$1.52{\cdot}10^{-1}$	$2.67{\cdot}10^{-7}$	0.98	$5.95{\cdot}10^{-10}$

Table S4. Kinetic parameters: B is the power law amplitude (s^{α}), exponent α, A is the exponential amplitude and τ is the time constant (s), obtained from power law plus exponential fitting monitored at 470 nm upon pulsed 355 nm excitation for the **BVO|SiO|Ru$_{Med}$** photoanode at different applied potentials in a μ = 1 M pH 7 NaPi aqueous solution.

**E_{app} (vs NHE)**	**B (s^{α})**	**A**	**A**	**τ (s)**	**R^2**	**Chi-Sq**
**0 V**	$8.20{\cdot}10^{-5}$	0.53	$8.04{\cdot}10^{-2}$	$6.18{\cdot}10^{-7}$	0.99	$4.41{\cdot}10^{-10}$
**0.2 V**	$9.73{\cdot}10^{-5}$	0.52	$6.47{\cdot}10^{-2}$	$5.51{\cdot}10^{-7}$	0.99	$5.46{\cdot}10^{-10}$
**0.4 V**	$1.02{\cdot}10^{-4}$	0.51	$6.21{\cdot}10^{-2}$	$5.65{\cdot}10^{-7}$	0.99	$3.59{\cdot}10^{-10}$
**0.6 V**	$1.30{\cdot}10^{-4}$	0.50	$5.07{\cdot}10^{-2}$	$5.16{\cdot}10^{-7}$	0.99	$7.23{\cdot}10^{-10}$
**0.8 V**	$2.00{\cdot}10^{-4}$	0.47	$1.50{\cdot}10^{-1}$	$4.74{\cdot}10^{-7}$	0.98	$6.43{\cdot}10^{-10}$
**1 V**	$1.96{\cdot}10^{-4}$	0.46	$2.30{\cdot}10^{-1}$	$4.90{\cdot}10^{-7}$	0.99	$1.03{\cdot}10^{-9}$

Table S5. Kinetic parameters: B is the power law amplitude (s^{α}), exponent α, A is the exponential amplitude and τ is the time constant (s), obtained from power law plus exponential fitting monitored at 470 nm upon pulsed 355 nm excitation for the **BVO|SiO|Ru_{Woc}-Ru_{Med}** photoanode at different applied potentials in a μ = 1 M pH 7 NaPi aqueous solution.

E_{app} (vs NHE)	B (s^{α})	α	A	τ (s)	R^2	Chi-Sq
**0 V**	$7.70 \cdot 10^{-5}$	0.53	$9.29 \cdot 10^{-2}$	$3.09 \cdot 10^{-7}$	0.96	$4.90 \cdot 10^{-9}$
**0.2 V**	$5.15 \cdot 10^{-5}$	0.52	$1.41 \cdot 10^{-2}$	$2.94 \cdot 10^{-7}$	0.97	$2.62 \cdot 10^{-9}$
**0.4 V**	$1.09 \cdot 10^{-4}$	0.50	$1.75 \cdot 10^{-1}$	$2.58 \cdot 10^{-7}$	0.95	$2.90 \cdot 10^{-9}$
**0.6 V**	$1.63 \cdot 10^{-4}$	0.47	$1.62 \cdot 10^{-1}$	$2.26 \cdot 10^{-7}$	0.96	$2.90 \cdot 10^{-9}$
**0.8 V**	$2.24 \cdot 10^{-4}$	0.46	$1.52 \cdot 10^{-1}$	$2.04 \cdot 10^{-7}$	0.98	$6.02 \cdot 10^{-9}$
**1 V**	$2.05 \cdot 10^{-4}$	0.46	$1.83 \cdot 10^{-1}$	$1.74 \cdot 10^{-7}$	0.98	$1.03 \cdot 10^{-9}$

Table S6. Comparison of the lifetimes of the photogenerated trapped holes of several literature reported metal oxides and molecular catalyst anchored on BVO photoanodes with their respective blanks.

Photoanode	Laser exc.[a] (nm)	Laser mon.[b] (nm)	E_{app}[c] (V vs RHE)	Lifetime[d] (s)	Loading (mol/cm^2)[e]	Electrolyte[f]	pH	Ref
**$BiVO_4$|LDH**	355	540	1.23	$1.40 \cdot 10^{-6}$	-	1 M KBi	9	3
**$BiVO_4$|LDH|$CoPO_3$**	355	540	1.23	$7.40 \cdot 10^{-7}$	-	1 M KBi	9	3
**$BiVO_4$|pGO|LDH|$CoPO_3$**	355	540	1.23	$2.30 \cdot 10^{-7}$	-	1 M KBi	9	3
**WO_3|$BiVO_4$**	355	470	1.23	$3.00 \cdot 10^{-6}$	0	0.1 M NaPi	7	4
**WO_3|$BiVO_4$|$CoFeO_x$**	355	470	1.23	$2.00 \cdot 10^{-7}$	-	0.1 M NaPi	7	4
**WO_3|$BiVO_4$|CoFe-PB**	355	470	1.23	$8.00 \cdot 10^{-7}$	-	0.1 M NaPi	7	4
**BVO**	355	470	1.23	$2.92 \cdot 10^{-6}$	0	1 M NaPi	7	Tw
**BVO|SiO|Ru_{Woc}**	355	470	1.23	$2.38 \cdot 10^{-7}$	$2.2 \cdot 10^{-11}$	1 M NaPi	7	Tw
**BVO|SiO|Ru_{Woc}-Ru_{Med}**	355	470	1.23	$2.04 \cdot 10^{-7}$	$1.6 \cdot 10^{-9}$	1 M NaPi	7	Tw

[a] Laser excitation; [b] Laser monitoring; [c] Applied potential (V vs. RHE) is the potential exerted on stability test; [d] Lifetime at 1.23 V vs. RHE; [e] Loading of catalyst anchored onto the photoanode; [f] NaPi: sodium phosphate, KPi: potassium phosphate, KBi: potassium borate.

Photoelectrochemical measurements

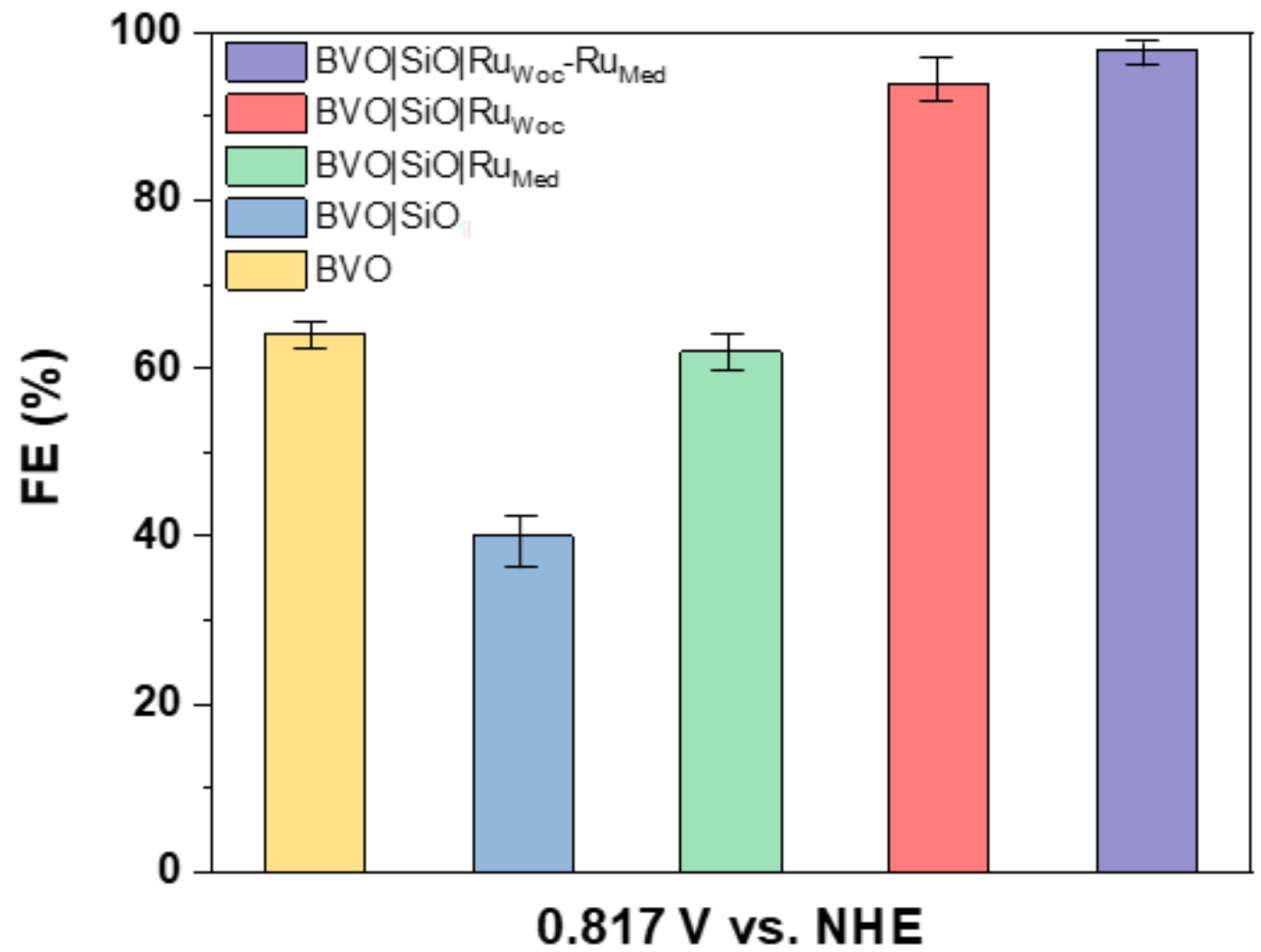

Figure S16. Bar chart showing the Faradaic efficiencies based on the amount of dioxygen generated (measured with a Clark electrode), achieved during the CPEs in μ = 1.0 M pH 7 phbf aqueous solution under 1 sun illumination at E_{app} = 0.817 V vs. NHE for 1 hour, for: **BVO** (yellow trace), **BVO|SiO** (blue), **BVO|SiO|Ru$_{Med}$** (green), **BVO|SiO|Ru$_{Woc}$** (red) and **BVO|SiO|Ru$_{Woc}$-Ru$_{Med}$** (purple) described in detail in Figure 4b in the main MS. RE, Hg/HgSO$_4$ (K$_2$SO$_4$ sat.); CE, Pt coil.

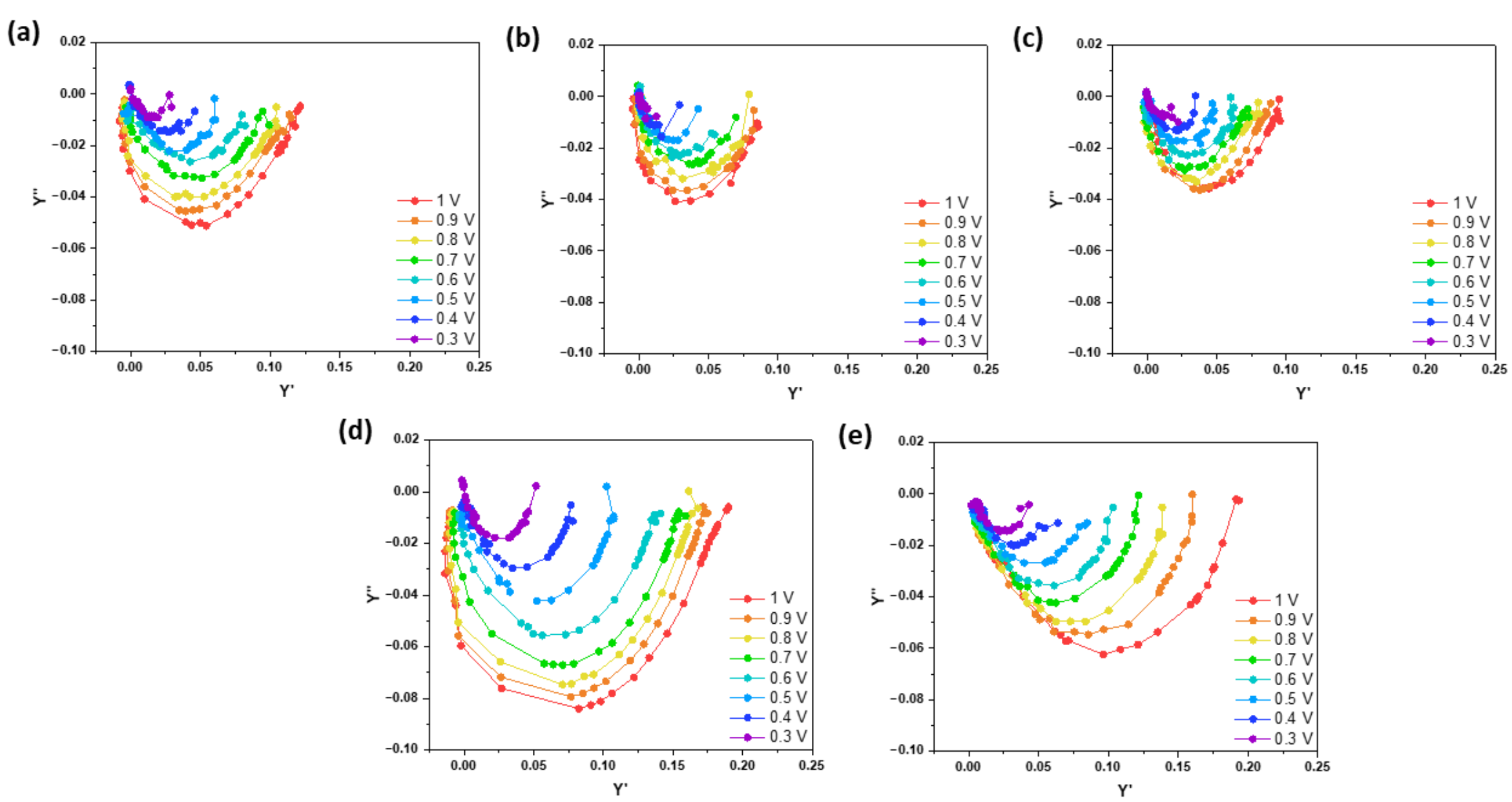

Figure S17. IMPS spectra of (a) **BVO**, (b) **BVO|SiO**, (c) **BVO|SiO|Ru$_{Med}$**, (d) **BVO|SiO|Ru$_{Woc}$** and (e) **BVO|SiO|Ru$_{Woc}$-Ru$_{Med}$** at the indicated applied potentials in a 1 M pH 7 phbf aqueous solution with 365 nm excitation, a Ag/AgCl (3M KCl) reference electrode and a Pt mesh counter electrode.

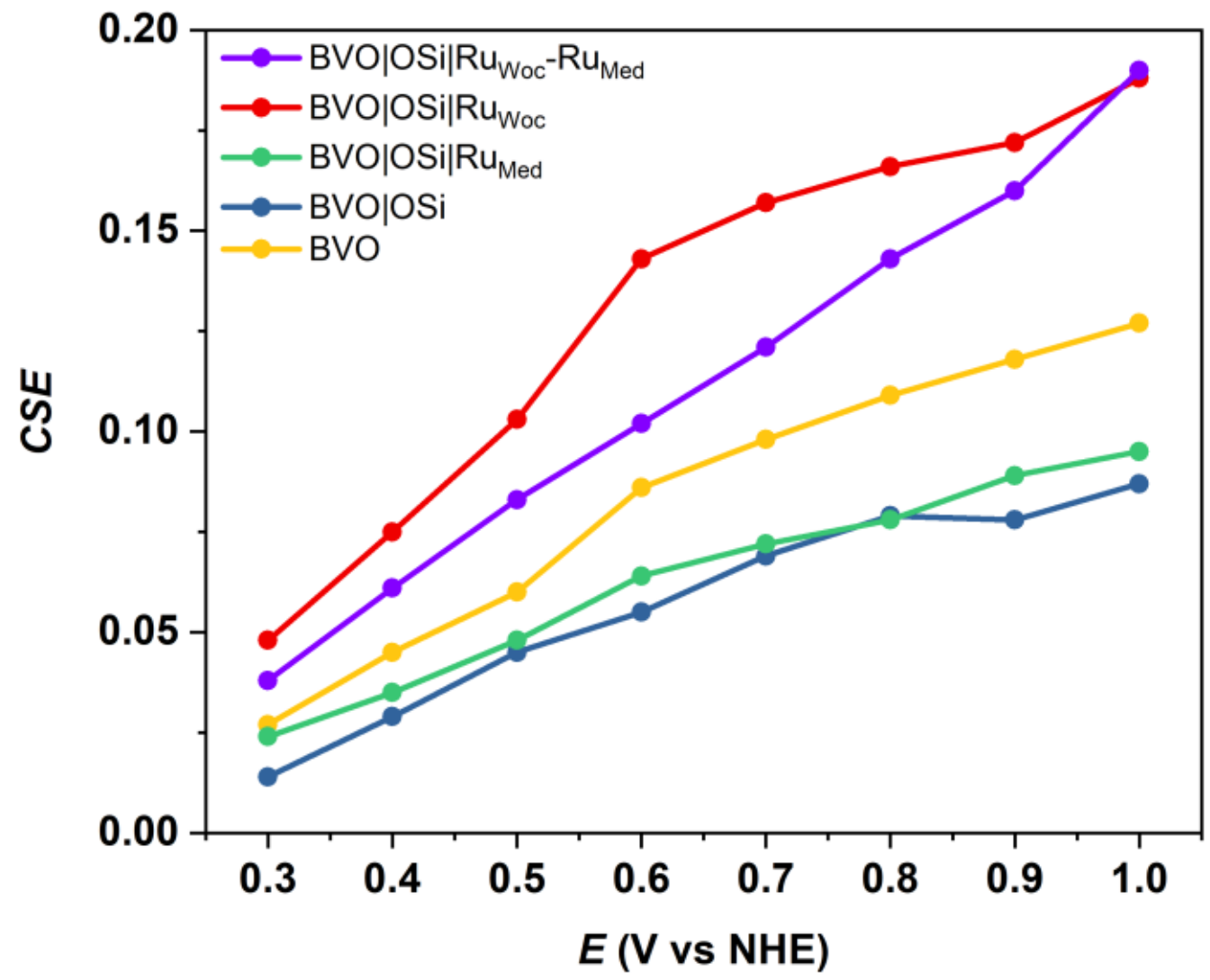

Figure S18. Charge Separation Efficiency (CSE) values for **BVO** (yellow trace), **BVO|SiO** (blue trace), **BVO|SiO|Ru$_{Med}$** (green trace), **BVO|SiO|Ru$_{Woc}$** (red trace) and **BVO|SiO|Ru$_{Woc}$-Ru$_{Med}$** (purple trace). The measurements were performed in a μ = 1 M pH 7 phbf aqueous solution. Data extracted from Figure S17.

Table S7. Charge Separation Efficiency (CSE) values obtained from the IMPS spectra for the photoanodes: **BVO**, **BVO|SiO**, **BVO|SiO|Ru$_{Med}$**, **BVO|SiO|Ru$_{Woc}$** and **BVO|SiO|Ru$_{Woc}$-Ru$_{Med}$**, as a function of applied potential.

**E_{app}[a] (columns) / Photoanode (rows)**	**0.3 V**	**0.4 V**	**0.5 V**	**0.6 V**	**0.7 V**	**0.8 V**	**0.9 V**	**1 V**
**BVO**	0.027	0.045	0.060	0.086	0.098	0.109	0.118	0.127
**BVO|SiO**	0.014	0.029	0.045	0.055	0.069	0.079	0.078	0.087
**BVO|SiO|Ru$_{Med}$**	0.024	0.035	0.048	0.064	0.072	0.078	0.089	0.095
**BVO|SiO|Ru$_{Woc}$**	0.048	0.075	0.103	0.142	0.157	0.166	0.172	0.188
**BVO|SiO|Ru$_{Woc}$-Ru$_{Med}$**	0.038	0.061	0.083	0.102	0.121	0.143	0.161	0.190

[a] *Applied potential (V vs NHE).*

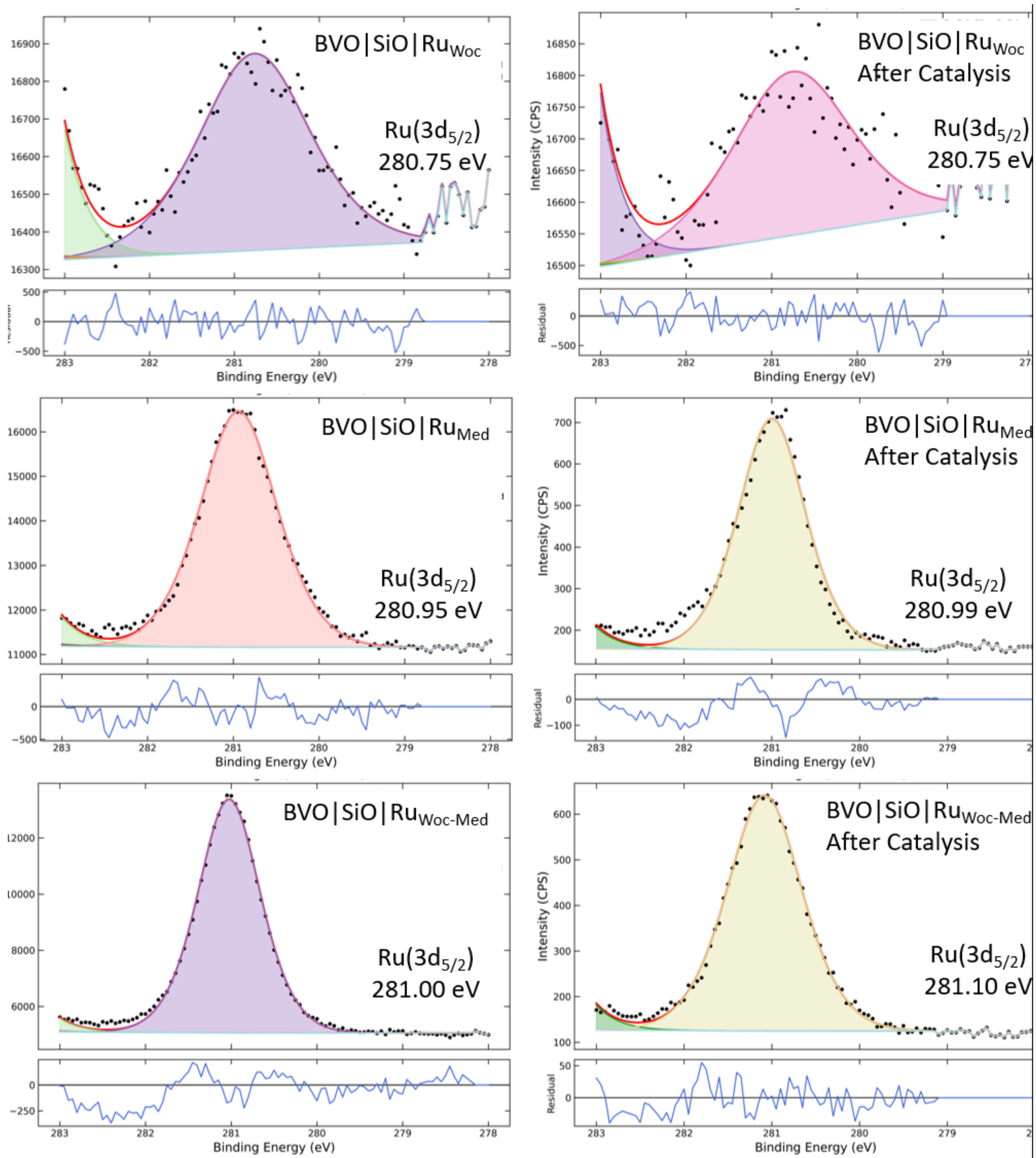

Figure S19. Ru $3d_{5/2}$ XPS analysis for **BVO|SiO|Ru$_{Woc}$, BVO|SiO|Ru$_{Med}$** and **BVO|SiO|Ru$_{Woc}$-Ru$_{Med}$** before and after CPE in μ = 1.0 M pH 7 phbf aqueous solution under 1 sun illumination at E_{app} = 0.817 V vs. NHE for 1 hour (experiment described in Figure 4b). The results show that the band associated with the Ru($3d_{5/2}$) does not undergo any significant changes, indicating that the nature of the catalyst is preserved under the catalytic conditions.

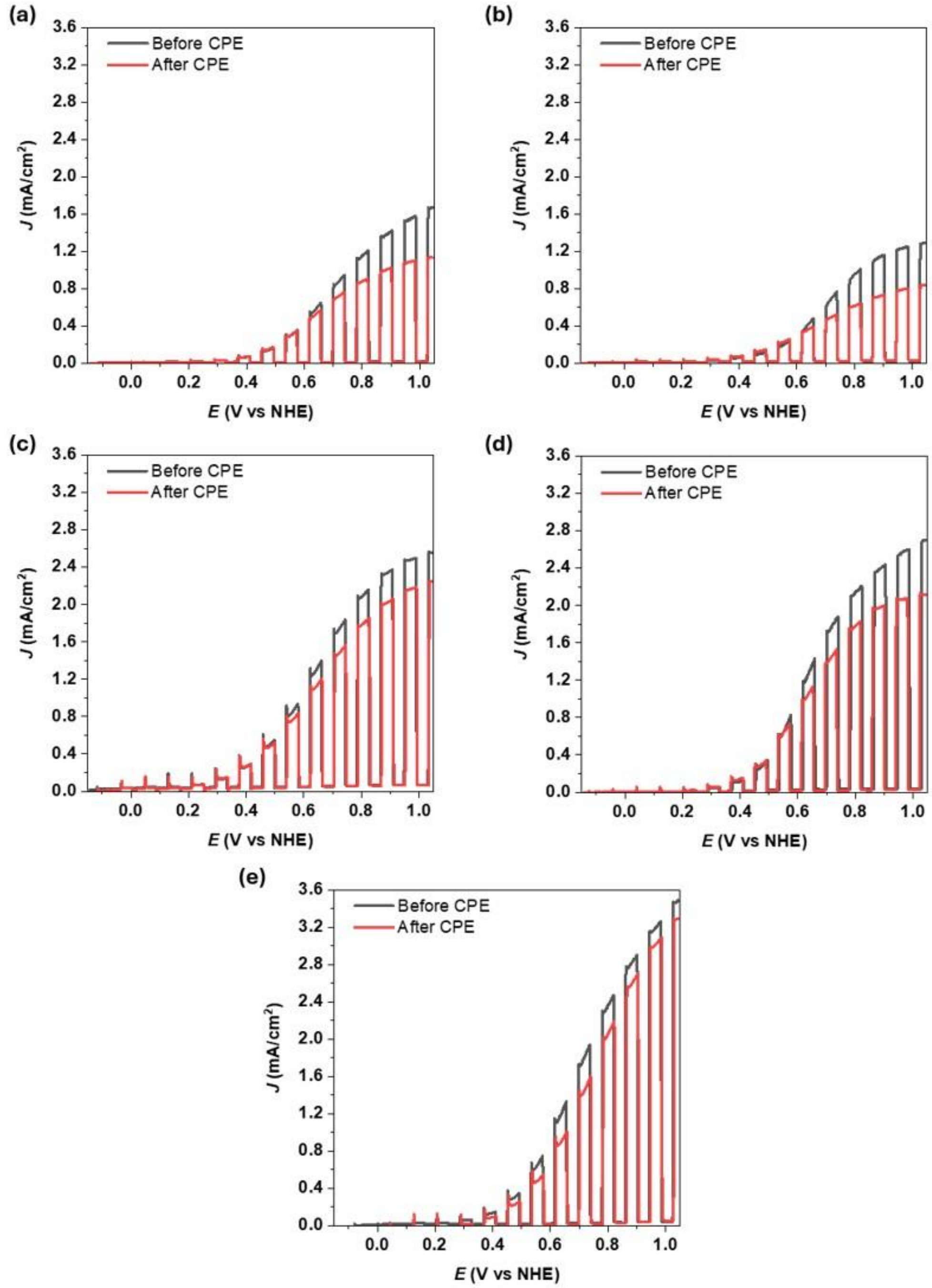

Figure S20. Chopped linear sweep voltammetry at 10 mV/s irradiated under 1 sun illumination (100 mW/cm^2) for 3 s on/off cycles for: (a) **BVO**, (b) **BVO|SiO**, (c) **BVO|SiO|Ru$_{Med}$**, (d) **BVO|SiO|Ru$_{Woc}$** and (e) **BVO|SiO|Ru$_{Woc}$-Ru$_{Med}$** in a μ = 1.0 M pH 7 phbf aqueous solution before (black trace) and after (red trace) bulk photoelectrolysis at E_{app} = 0.82 V vs. NHE for 1 h (experiment described in Figure 4b main MS).

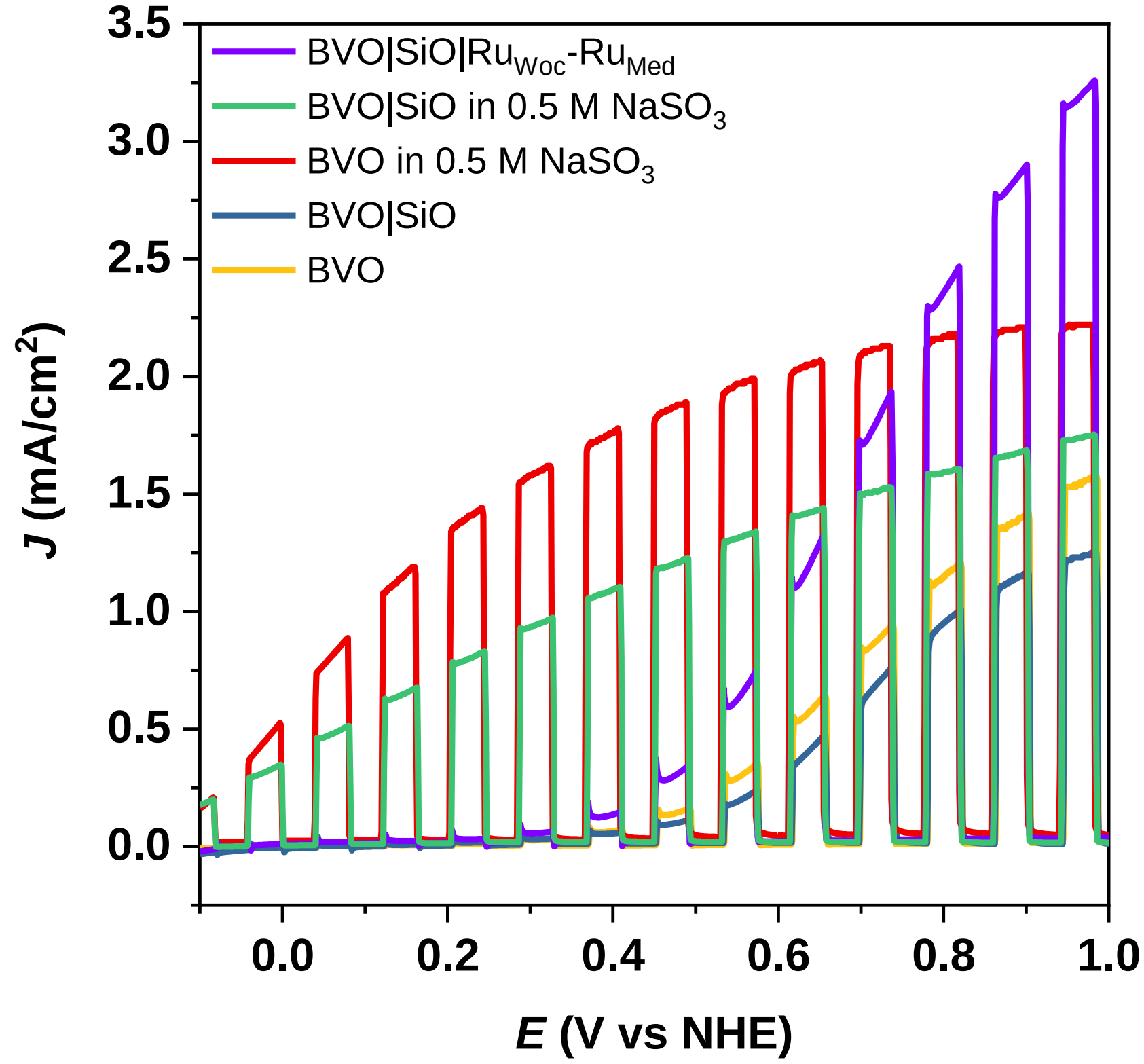

Figure S21. Chopped linear sweep voltammetry at 10 mV/s irradiated under 1 sun illumination (100 mW/cm^2) for 3 s on/off cycles for: **BVO** (yellow trace), **BVO** in 0.5 M Na_2SO_3 (red), **BVO|SiO** (blue), **BVO|SiO** in 0.5 M Na_2SO_3 (green) and **BVO|SiO|Ru$_{Woc}$-Ru$_{Med}$** (purple), measured in a μ = 1.0 M pH 7 phbf aqueous solution. RE, Hg/$HgSO_4$ (K_2SO_4 sat.); CE, Pt coil.

References

(1) Tris-Marzo, P.; Veclani, D.; Venturini, A.; Martínez-Belmonte, M.; Gil-Sepulcre, M.; Llobet, A. Molecular Anodes for Electrocatalytic Water Oxidation Based on Self-Assembled Bilayers Driven by Electron Transfer Mediators. *J. Am. Chem. Soc*. **2025**, *147* (15), 12686–12695. https://doi.org/10.1021/jacs.5c00489.

(2) Baek, J. H.; Kim, B. J.; Han, G. S.; Hwang, S. W.; Kim, D. R.; Cho, I. S.; Jung, H. S. $BiVO_4/WO_3/SnO_2$ Double-Heterojunction Photoanode with Enhanced Charge Separation and Visible Transparency for Bias-Free Solar Water Splitting with a Perovskite Solar Cell. *ACS Appl. Mater. Interfaces* **2017**, *9* (2), 1479–1487. https://doi.org/10.1021/acsami.6b12782.

(3) Ye, S.; Ding, C.; Chen, R.; Fan, F.; Fu, P.; Yin, H.; Wang, X.; Wang, Z.; Du, P.; Li, C. Mimicking the Key Functions of Photosystem II in Artificial Photosynthesis for Photoelectrocatalytic Water Splitting. *J. Am. Chem. Soc.* **2018**, *140* (9), 3250-3256. https://doi.org/10.1021/jacs.7b10662.

(4) Vecchi, P.; Ruani, F.; Mazzanti, M.; Loague, Q. R.; Mazzaro, R.; Boscherini, F.; Ventura, B.; Meyer, G. J.; Armaroli, N.; Caramori, S.; Pasquini, L. Impact of Co-Fe Overlayers on Charge Carrier Dynamics at $WO_3/BiVO_4$ Heterojunctions: A Picosecond-to-Second Spectroscopic Analysis. *ACS Energy Lett*. **2024**, *9* (5), 2193-2200. https://doi.org/10.1021/acsenergylett.4c00650.
